# Advection versus diffusion in brain ventricular transport

**DOI:** 10.1186/s12987-025-00692-3

**Published:** 2025-08-13

**Authors:** Halvor Herlyng, Ada J. Ellingsrud, Miroslav Kuchta, Inyoung Jeong, Marie E. Rognes, Nathalie Jurisch-Yaksi

**Affiliations:** 1 Department of Numerical Analysis and Scientific Computing, Simula Research Laboratory, Oslo, Norway; 2 Department of Clinical and Molecular Medicine, Norwegian University of Science and Technology, Trondheim, Norway; 3 K. G. Jebsen Center for Brain Fluid Research, Oslo, Norway

**Keywords:** Cerebrospinal fluid, Brain ventricles, Cilia, Transport, Finite elements

## Abstract

Cerebrospinal fluid (CSF) is integral to brain function. CSF provides mechanical support for the brain and helps distribute nutrients, neurotransmitters and metabolites throughout the central nervous system. CSF flow is driven by several processes, including the beating of motile cilia located on the walls of the brain ventricles. Despite the physiological importance of CSF, the underlying mechanisms of CSF flow and solute transport in the brain ventricles remain to be comprehensively resolved. This study analyzes and evaluates specifically the role of motile cilia in CSF flow and transport. We developed finite element methods for modeling flow and transport using the geometry of embryonic zebrafish brain ventricles, for which we have detailed knowledge of cilia properties and CSF motion. The computational model is validated by in vivo experiments that monitor transport of a photoconvertible protein secreted in the brain ventricles. Our results show that while cilia contribute to advection of large particles, diffusion plays a significant role in the transport of small solutes. We also demonstrate how cilia location and the geometry of the ventricular system impact solute distribution. Altogether, this work presents a computational framework that can be applied to other ventricular systems, together with new concepts of how molecules are transported within the brain and its ventricles.

## Introduction

Brain development and function depend on numerous regulatory processes, including the flow of cerebrospinal fluid (CSF) [1]. Produced predominantly by the choroid plexus, CSF is a clear liquid that flows through the ventricular system and subarachnoid space [2–4]. By transporting growth factors, nutrients, neurotransmitters and metabolites, as well as removing waste products, CSF regulates brain development and homeostasis [1, 5, 6]. Disruption of CSF circulation is detrimental to brain health and disturbs cognitive and motor functions [7], and accumulation of CSF leads to hydrocephalus, a disorder often requiring emergency surgical treatment [8–11].

The flow of CSF in the ventricular system is driven by multiple physiological processes, including the beating motion of motile cilia, CSF secretion by the choroid plexus, and pulsatile pressure gradients generated by the cardiac cycle, respiration, neural activity, and bodily movement [2, 5, 6, 12–17]. Cilia are hair-like structures found in a vast diversity of biological organisms and in several human organs, including the brain [5, 18–29]. Notably, motile cilia are found on the ventricular surface of the brain, known as the ependyma, and beat in a coordinated pattern at frequencies of 10–40 Hz to mediate fluid flow [5, 23, 25, 30, 31]. Given their microscopic size, ranging from 5 to 15 µm, cilia contribute primarily to flow near the ependyma in the brain [5, 14, 32, 33]. In contrast, cardiac, respiratory and delta waves at lower frequencies induce pulsatile flow at larger scales in the brain ventricular system, subarachnoid space, and perivascular spaces [12, 16, 34–37].

To date, it remains poorly understood how motile cilia contribute to the overall fluid flow and solute transport within the brain. Experimental approaches face technical challenges: the microscopic size and relatively high beating frequencies of the cilia [14, 23, 38–41], as compared to other slower fluid dynamic processes that span larger spatial scales [12, 16, 34, 36], make cilia difficult to monitor in vivo in the rodent and human brain. Until now, most studies have analyzed one physical mechanism at a time, especially in mammals. For instance, studies using brain explants have convincingly shown that cilia generate complex flow patterns at the surface of the brain ventricles [33, 42, 43]; however, they did not study the role of other contributions to CSF flow and the accompanying transport, such as pressure gradients associated with respiration and cardiac pulsations. Moreover, while MRI technology can measure CSF motion in humans [44–46], it cannot detect the microscopic movements of cilia to understand their impact in the large human brain [32]. These technical limitations of studying cilia-mediated flow and transport make mathematical modeling and computational investigations an attractive complement to experimental studies. Previous simulation studies have focused on modeling the interaction between single or several cilia and surrounding fluids [47–51]. Others considered cilia-mediated flow in the brain [29, 32, 52, 53], and other applications such as ciliary transport of mucous and particles in airways [19, 31, 54], propulsion and locomotion of ciliated organisms [21, 22, 27], and oxygen regulation in coral reefs [20]. These simulation studies have provided valuable insights on how cilia mediate complex biological flows. However, we still lack a comprehensive understanding of how cilia contribute to CSF motion and solute transport within the brain. This is of particular importance since disorders such as hydrocephalus are observed in animal models and humans with deficient motile cilia [14, 55–57], indicating that cilia play a crucial role for healthy brain function.

In this study, we combined computational and experimental approaches to investigate the role of motile cilia in brain ventricular CSF flow and transport. We model the flow and transport by partial differential equations and solve them numerically with finite element methods to compute flow patterns and solute concentration dynamics. A geometry representing the embryonic zebrafish brain ventricles was used, for which we have detailed knowledge of cilia properties and CSF motion velocities and frequencies [14]. Notably, this early developmental stage allows us to simplify our model by omitting CSF secretion, which starts at later stages [58], while still being well-conserved with mammals [5, 14, 18, 41, 59]. The computational model is validated against experimental data by monitoring in vivo the dynamics of a photoconvertible fluorescent protein Dendra2 expressed within the brain ventricles. Our results show that diffusion plays a major role in the transport of small solutes, whereas cilia are paramount for the movement of larger particles. We also found that the location of cilia, as well as changes to the ventricular geometry, largely impact solute distribution. In summary, our work presents a computational framework that can be applied to other ventricular systems in the future, together with new concepts of how cilia distribute molecules and particles within the brain ventricles.

## Results

### Motile cilia induce flow compartmentalization within the brain ventricles

**Fig. 1:**
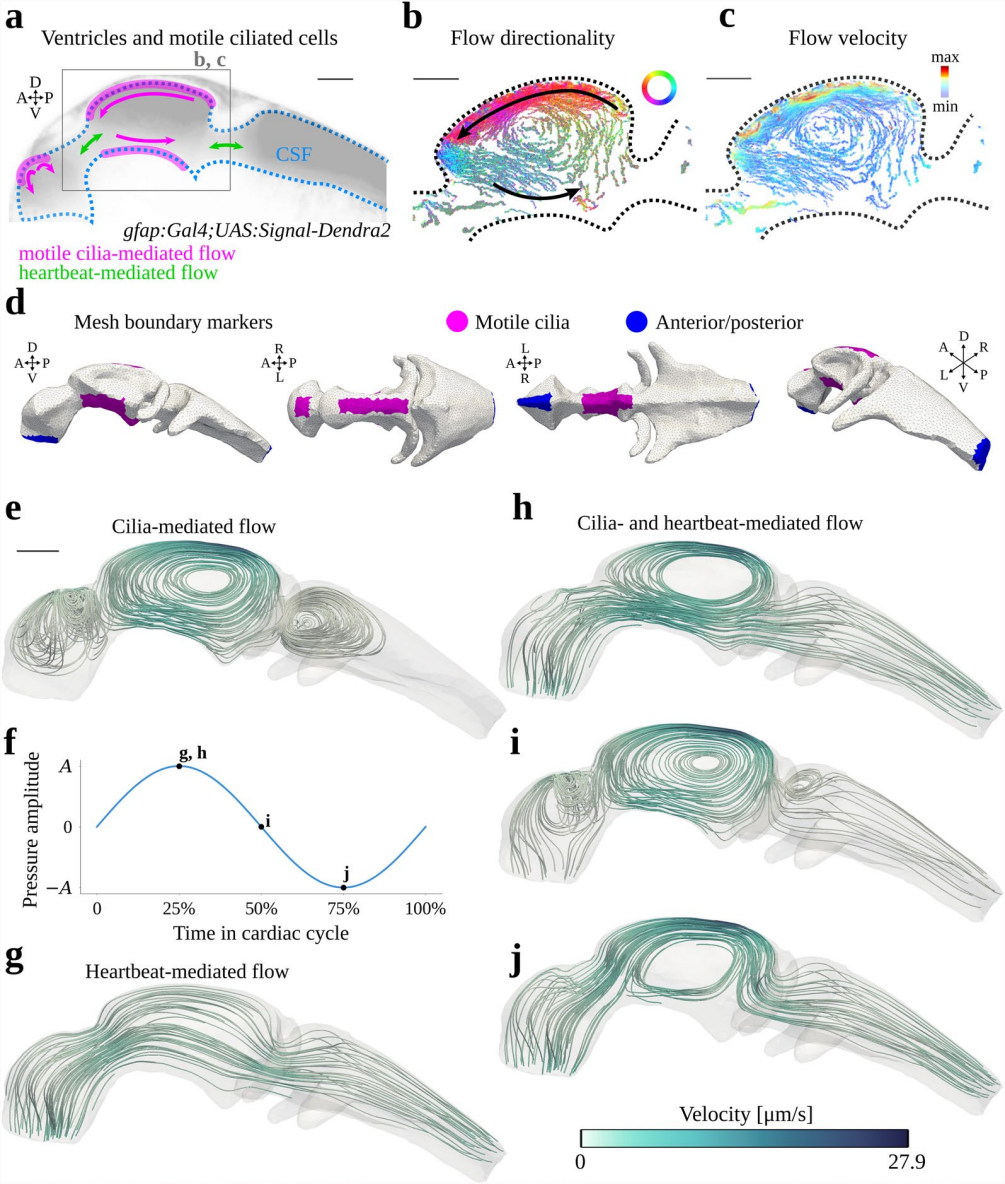
**a** Schematic illustration of the two cerebrospinal fluid (CSF) flow components modeled, motile cilia and cardiac pulsations, and their contributions to the flow patterns. **b**, **c** Particle tracking data visualizing CSF flow directionality (**b**) and velocity magnitudes (**c**) in the middle ventricle. **d** Computational mesh with marked facets indicating regions of motile cilia (magenta) and anterior/posterior facets (blue). Dorsal (D), ventral (V), anterior (A), posterior (P), left (L) and right (R) coordinates indicated. **e** Streamlines of the (steady-state) CSF flow field simulated with the cilia-driven/no-cardiac flow model. **f** Illustration of the cardiac-related normal pressure forcing imposed on the anterior/posterior boundaries. Time instants of streamline plots indicated. **g** Streamlines of the CSF flow field simulated with the cardiac-induced/no-cilia flow model at a time 25% into the cardiac cycle. **h–j** Streamlines simulated with the baseline flow model (cilia+cardiac) at a time 25% (**h**), 50% (**i**) and 75% (**j**) into the cardiac cycle. We remark that the velocities in the cardiac-induced/no-cilia flow model are one order of magnitude lower (maximum 4.8 µm/s) than the velocities in the flow models that include motile cilia (maxima 26.7 µm/s (cilia-driven/no-cardiac) and 27.9 µm/s (baseline)). Scale bars 50 µm.

In vivo particle tracking reveals characteristic CSF flow patterns in embryonic zebrafish brain ventricles (Fig. 1a–c) [14]. We attempt to accurately represent these flow patterns with computational fluid dynamics driven by cardiac pulsations and motile cilia lining the surface of the brain ventricles, using a computational geometry generated from confocal imaging data of a zebrafish embryo (Fig. 1d). A description of the geometry is provided in the Methods section.

Assuming that viscous forces are dominating the ventricular CSF flow, we model the flow by the Stokes equations. The net forces of motile cilia acting on the CSF are modeled as a steady, bidirectional tangential traction acting on parts of the ventricular wall (Fig. 1d), with an impermeability condition (no normal flow) at the walls. Simulations show that this traction alone leads to a partial compartmentalization of the ventricular system, with large-scale vortex structures in the anterior, middle and posterior ventricles (Fig. 1e). The highest flow speeds (26.7 µm/s) occur in the vicinity of the dorsal cilia in the middle ventricle. To model the cardiac pulsations, we impose a pulsatile pressure difference between the anterior and posterior boundaries (Fig. 1d, f). The resulting flow is pulsatile and directional (Fig. 1g), and in the absence of the cilia, the vortex structures vanish. The velocity magnitude peaks at 4.8 µm/s. CSF initially flows rostrocaudally before changing direction to caudorostral in the middle of the cardiac cycle.

When combining cilia motion and cardiac pulsations, we recognize flow features persisting throughout the cardiac cycle, now with vortex structures in the anterior and middle ventricles and directional flow in the posterior ventricle (Fig. 1h–j, Supplementary Video S1). These characteristic patterns suggest that the flow in the anterior and middle ventricles is primarily driven by the motile cilia, while the cardiac pressure pulsations dominate within the posterior ventricle and in the ducts connecting the ventricular compartments. The CSF flow speed peaks at the time of peak caudal cardiac pulsatile flow, when both the tangential traction induced by the cilia and the pulsatile pressure-driven flow are aligned with the rostrocaudal axis, reaching up to 27.9 µm/s dorsally in the posterior part of the middle ventricle. The flow Reynolds number is $\text{Re} \approx 0.004$, justifying the assumption of Stokes flow. In summary, the computational CSF flow model reproduces the flow features observed in vivo [14].

### Ventricular solute transport is advection-dominated for larger molecules

**Fig. 2:**
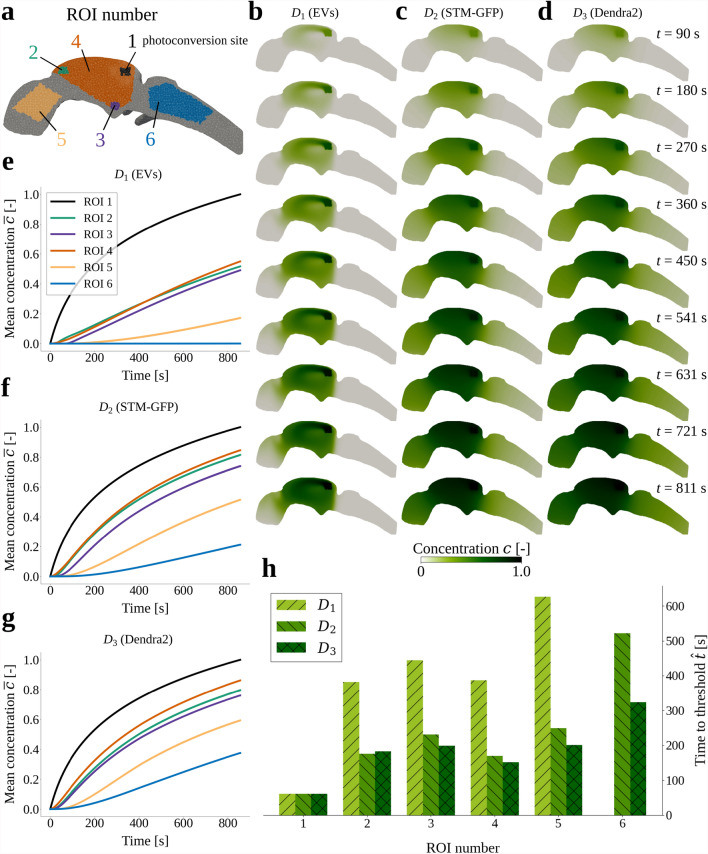
Simulated distribution and evolution after photoconversion of extracellular vesicles (EVs, $D_1$), STM-GFP ($D_2$) and Dendra2 ($D_3$). **a** Geometry-centered clip of the ventricles mesh showing the locations of the regions of interest (ROIs). **b–d** Slices of the concentration field simulated with diffusion coefficients $D_1$, $D_2$ and $D_3$ for nine time instants covering the time range from 90 s to 811 s, corresponding to 200 to 1800 cardiac cycles, for even intervals of 200 cycles. Slices are taken in the *xz*-plane ($y=0.140$ mm, the center of the geometry). **e–g** Mean concentration $\overline{c}$ in each ROI as function of time for diffusion coefficients $D_1$, $D_2$ and $D_3$. **h** The time $\hat{t}$ before the mean concentration $\overline{c}$ exceeds a threshold value of 0.25 (ROIs 2-4) or 0.10 (ROIs 5, 6) in each simulation setup. Note that there is no bar for $D_1$ in ROI 6, because the threshold value was never exceeded.

Proteins, neurotransmitters and other molecules intrinsically diffuse within the brain ventricles, but are also advected by the flow of CSF. A natural question is which of these mechanisms dominates for any given type of solute. To address this question, we simulate the distribution and evolution of three different solutes injected within a region of interest (ROI 1) in the dorsal region of the middle ventricle (Fig. 2a) and subject to the CSF flow induced by motile cilia and cardiac pressure pulsations. In particular, we consider three different molecular diffusion coefficients, corresponding to a large extracellular vesicle (EV, $D_1 = 2.17 \times 10^{-12}$ $\text {m}^2$/s), a 90 kDa Starmaker-Green Fluorescent Protein (STM-GFP, $D_2 = 5.75 \times 10^{-11}$ $\text {m}^2$/s), and a 26 kDa photoconvertible protein Dendra2 ($D_3 = 1.15 \times 10^{-10}$ $\text {m}^2$/s).

Interestingly, the distribution patterns differ between the solutes. For the smallest diffusion coefficient, representing extracellular vesicles, the advective transport by the CSF dominates diffusion: the CSF flow structures are evident in the concentration field, indicating that transport along the streamlines is more rapid than the diffusion across the streamlines (Fig. 2b). The balance between diffusion and advection shifts with increasing diffusion coefficient as solute spreads more uniformly throughout the ventricular geometry (Fig. 2b–d). Notably, the smaller solutes with higher diffusion coefficients spread more quickly to distal regions. Computing Péclet numbers, we find $\text{Pe}_1=664$, $\text{Pe}_2=25$ and $\text{Pe}_3=13$ for $D_1$, $D_2$ and $D_3$, respectively. With all $\text{Pe} > 1$, this indicates that advection is the dominant transport mechanism on a global scale.

To further quantify the solute transport, we examine the mean concentrations $\overline{c}_i(t)$ in six ROIs over time (Fig. 2e–g) and, in addition, the times $\hat{t}_i$ when the mean concentrations first exceed a threshold value $\hat{c}_i$ (Fig. 2h). Higher diffusion coefficients result in more rapid transport (Fig. 2e–g), with lower times-to-threshold in ROIs 2–6 (Fig. 2h). For all three diffusion coefficients, the solute first spreads within the middle ventricle, covering ROIs 2–4, before appearing in the anterior ventricle (ROI 5) and finally reaching the posterior ventricle (ROI 6). Notably, the extracellular vesicles, associated with the smallest diffusion coefficient, do not reach the posterior ventricle (ROI 6) within the simulated time frame Fig. 2b, e.

### Model validation by comparison with photoconversion experiments

**Fig. 3:**
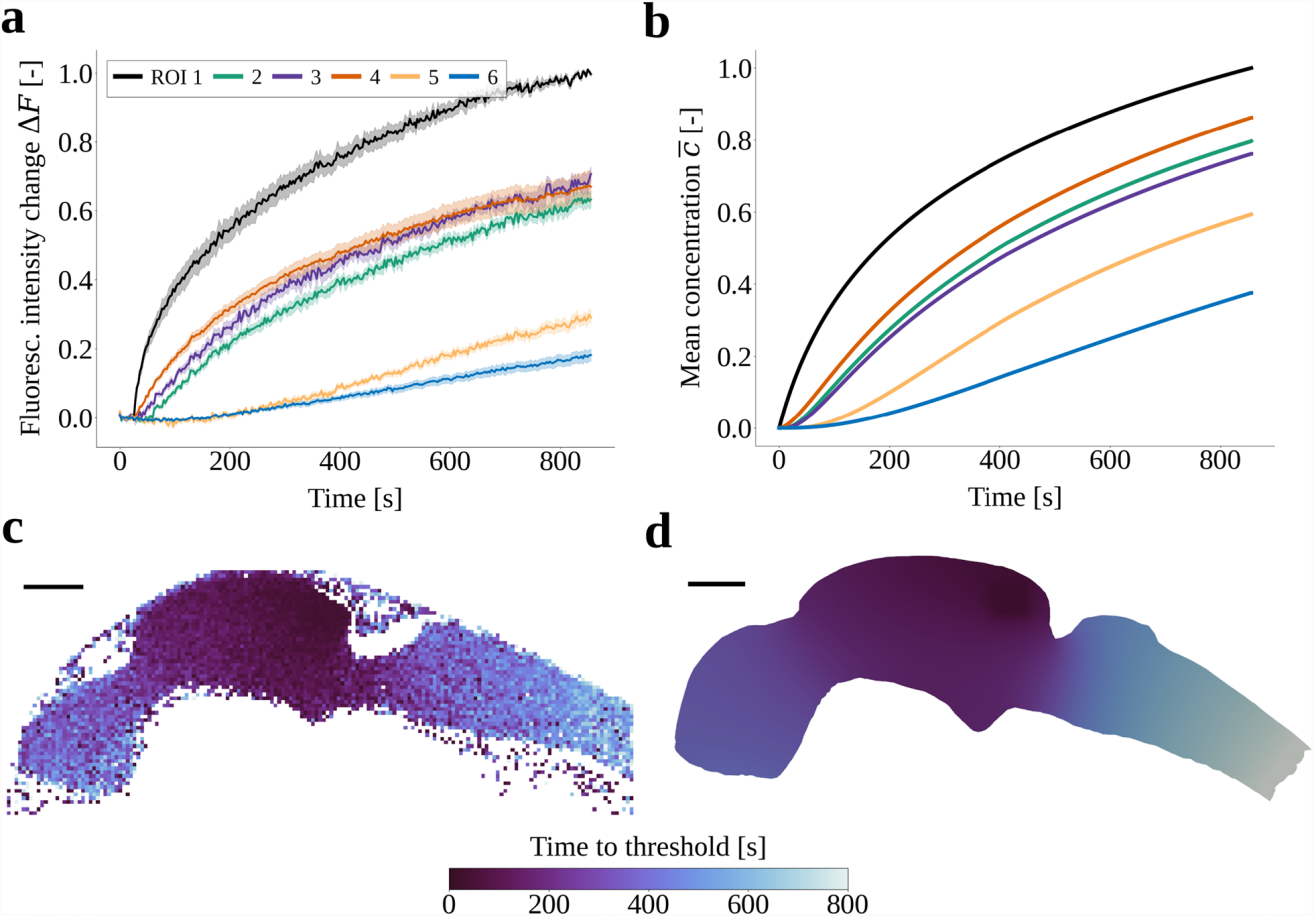
**a** Relative change in fluorescence intensity $\Delta F(t)$ in each region of interest (ROI) observed during experiments monitoring the movement of Dendra2 proteins photoconverted by laser targeting ROI 1. Bold lines are mean values of the cohort ($n=8$) and shaded regions cover one standard deviation. **b** Mean concentration $\overline{c}(t)$ in each ROI in a simulation of Dendra2 transport with the diffusion coefficient $D_3$. **c** Time to $\Delta F(t)$ reaches a threshold value of 0.25 in one representative zebrafish embryo. **d** Time to $\overline{c}(t)$ reaches a threshold value of 0.25 for a transport simulation with diffusion coefficient $D_3$. The posterior-most regions of the geometries are excluded, because $\Delta F$ and $\overline{c}$ never exceeded the threshold value of 0.25 in these regions. Scale bars 50 µm.

To validate the model predictions, we performed photoconversion experiments in transgenic zebrafish embryos expressing the secreted Dendra2 in their brain ventricles. The approach is inspired by earlier work where a photoconvertible protein was injected into the ventricle [60]. By using a transgenic system where the protein is directly secreted into the CSF, we avoid detrimental effects of intraventricular injection on CSF properties. Our photoconversion protocol consisted of the acquisition of a baseline fluorescence intensity $F_0$, before localized exposure to a UV laser (at ROI 1) that converts Dendra2 from a green- to a red-emitting fluorescent protein (Supplementary Video S2). To quantify the transport of photoconverted proteins, we measured and calculated the change in fluorescence intensity $\Delta F(t) = (F(t)-F_0)/F_0$ over time (Fig. 3a).

Comparison of the intensity changes with the simulated concentration profiles in ROIs 1–6 (Fig. 3a, b) shows that the model predictions agree well with the experimental measurements. We note minor differences however. The simulated values of $\overline{c}_i$ at the final time (0.80, 0.76, 0.86, 0.60 and 0.38 in ROIs 2–6, respectively) are generally higher than the corresponding experimental $\Delta F$ values (0.63, 0.71, 0.67, 0.29 and 0.18). Moreover, we observe differences in the distribution within the middle ventricle, especially with regard to ROIs 2 and 3. Overall, we note that the time to reach a 25% threshold agree well between the model predictions and experimental values (Fig. 3c, d).

### Absence of ciliary motion affects local solute distribution

**Fig. 4:**
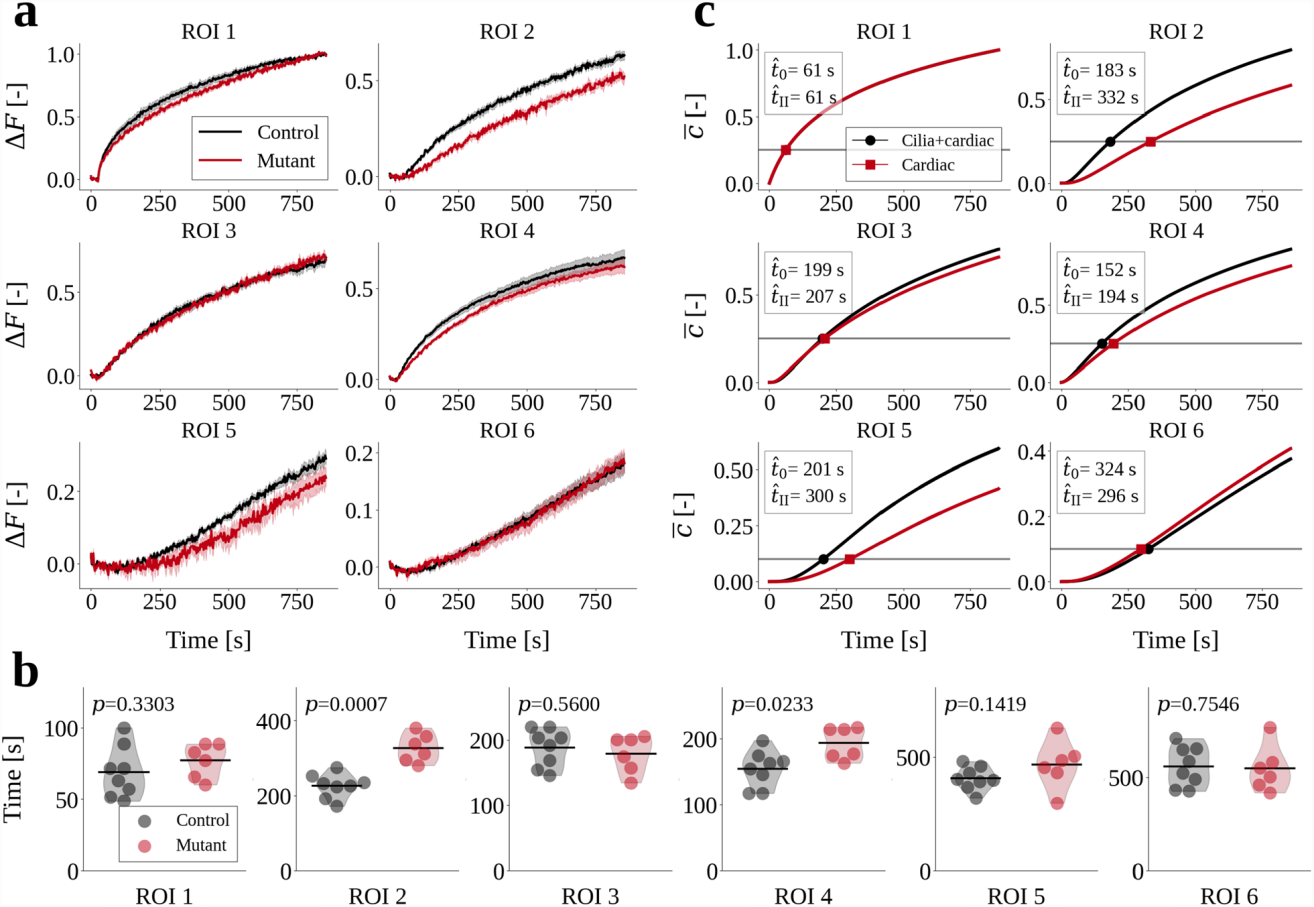
**a** Relative change in fluorescence intensity $\Delta F(t)$ in each region of interest (ROI) for control (black lines) and *smh* mutant (red lines) zebrafish. Bold lines are mean values of the cohorts and shaded regions cover one standard deviation. **b** Times when $\Delta F(t)$ first exceeds a threshold value, equal to 0.25 (ROIs 2–4) or 0.10 (ROIs 5, 6), for controls (gray dots) and *smh* mutants (red dots). The thick black lines denote mean values. The *p*-values were calculated using a Mann–Whitney U test with 95% confidence level, under the null hypothesis that the two distributions of control and mutant data are from the same population. **c** Mean concentration $\overline{c}(t)$ in each ROI, simulated using the baseline model (black lines) and the cardiac-induced/no-cilia model (red lines). The horizontal gray lines mark the threshold value $\hat{c}$, and the time $\hat{t}$ when the threshold is first exceeded is reported in the plot legends. Note that in panels **a** and **c** the vertical axes have different limits in each plot.

Ciliary motion is integral to physiological CSF flow, and motile-cilia deficiency has been associated with defective brain development and various pathologies in zebrafish, rodents and humans [14, 18, 33, 42, 55, 57, 61–66]. As observed both experimentally and computationally, absence of motile cilia alters CSF flow patterns Fig. 1g [14]. We next investigate the implications of these changes on brain ventricular transport. We experimentally measured the movement of proteins by monitoring photoconverted Dendra2 signal in control zebrafish and motile-cilia mutant *schmalhans* (*smh*, *ccdc103*) zebrafish [67]. In ROIs 2 and 4, which quantify Dendra2 distribution downstream of the photoconversion site, we observed a slower increase of fluorescence intensity (Fig. 4a): the mean times-to-threshold in the *smh* mutants were $45\%$ (ROI 2) and $25\%$ (ROI 4) higher compared to controls (Fig. 4b). We found no significant differences between *smh* mutants and controls in the Dendra2 distribution in ROIs 3, 5, and 6.

In the computational model, we represent the *smh* mutants by considering transport of Dendra2 with CSF flow driven by cardiac pressure pulsations only, representing the absence of motile cilia. Comparing the in silico mutants with transport predictions in the presence of both cardiac pulsations and motile cilia, we observe noticeably delayed dynamics in ROIs 2, 4 and 5, but little change for ROIs 3 and 6 (Fig. 4c). Compared to experimental data, the control and mutant scenarios differed more for the simulations, with the times-to-threshold in ROI 2 and 4 being 81% and 28% higher in the absence of cilia-mediated flow. Moreover, the predicted mean concentrations in ROI 5 deviate between the two scenarios, while in the experiments the difference in fluorescence intensities is not significant. Altogether, our findings identify that, for smaller proteins such as Dendra2, diffusion plays an important role for solute movement to regions upstream in the cilia-mediated flow fields or to distant ventricles, while the cilia affect the downstream mean concentrations.

### Regional loss of cilia motility delays transport

**Fig. 5:**
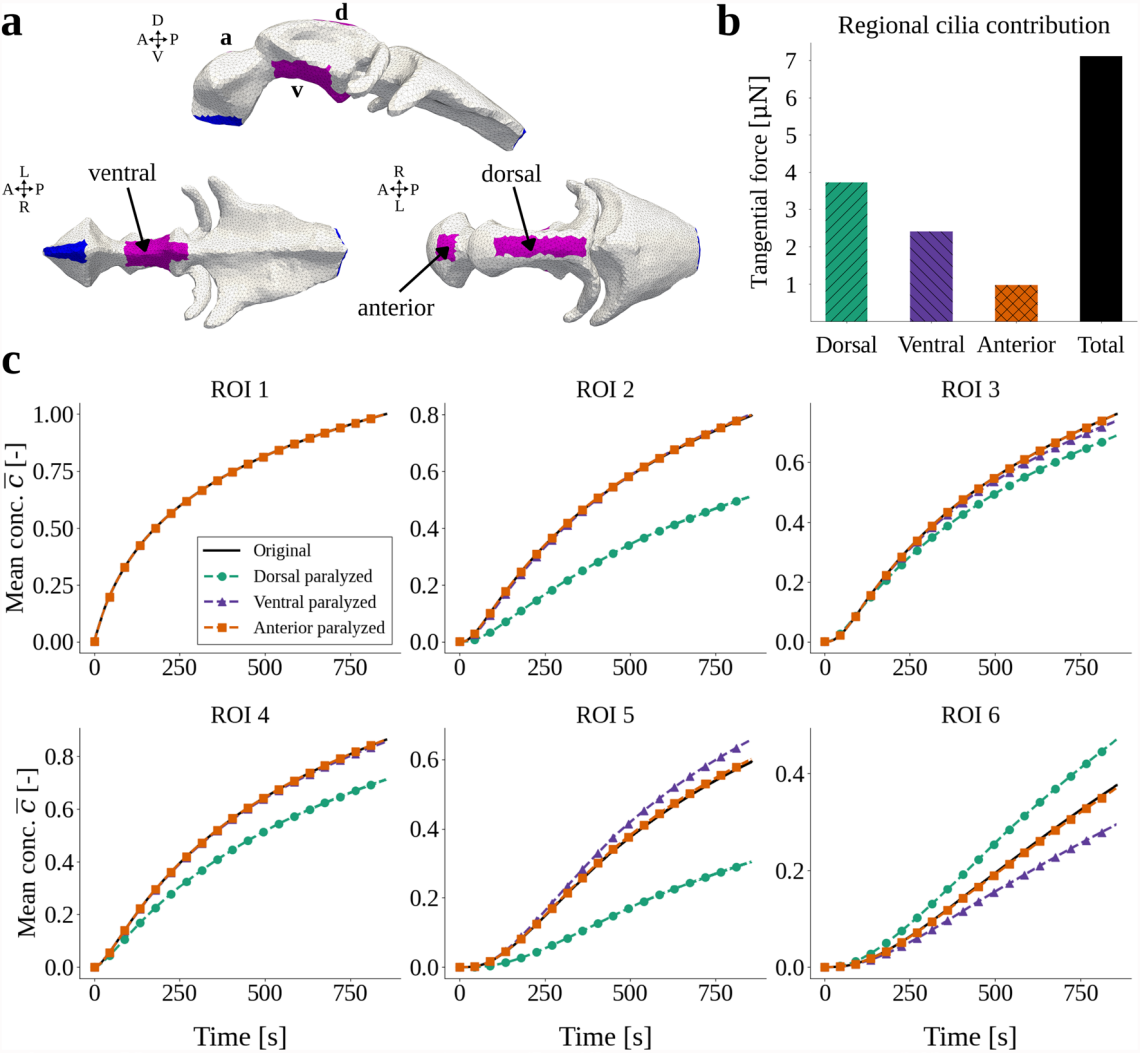
**a** The three separate cilia regions for which cilia paralysis is simulated by removing the cilia tangential traction boundary condition in the respective region. **b** The total force applied to the boundary with the cilia traction in each of the three separate ciliated regions, and the total force (the sum of the three regions), calculated with Equation (6). **c** Mean concentration $\overline{c}(t)$ for the six regions of interest (ROIs) in the three paralysis scenarios. The legend refers to the region where cilia are removed.

We previously characterized three distinct ciliated cell lineages in the embryonic zebrafish brain [14, 18]. To identify the effects of these lineages on CSF flow and transport, we investigate region-specific cilia paralysis with the computational model. Three cilia-covered regions were paralyzed separately: the anterior ventricle, and the dorsal and ventral parts of the middle ventricle (Fig. 5a). To simulate regional cilia paralysis, we remove the tangential traction exerted by the cilia in the respective region.

Removing cilia in the dorsal part of the middle ventricle has a strong effect on the CSF velocities: the maximum velocity magnitude drops from 27.9 µm/s to 7.9 µm/s. In vivo, these dorsal cilia generate the strongest flow at this developmental stage of the fish (Fig. 1c) [14]. In contrast, only removing cilia in the anterior ventricle or the ventral region of the middle ventricle has minimal impact, and results in maximum speeds of 27.9 µm/s and 27.4 µm/s, respectively. We remark that out of the total tangential forces, ventral cilia in the middle ventricle contribute by 34%, while the dorsal cilia exert 52% of the total tangential force (Fig. 5b). Thus, the CSF velocities are affected not only by the magnitude of the applied forces, but result from a nontrivial interplay between force directionality and location, and ventricular morphology.

Turning to transport of Dendra2, removal of the dorsal cilia again has the most pronounced effect. All mean concentration profiles (except ROI 1, where we impose the photoconversion curve) change noticeably (Fig. 5c). Compared to the baseline model results, we observe less solute distribution in the middle and anterior ventricles (ROIs 2–5), and more rapid transport to the posterior ventricle (ROI 6). On the other hand, removal of anterior cilia does not alter the mean concentrations (Fig. 5c). Finally, when removing ventral cilia in the middle ventricle more solute spreads towards the anterior ventricle (ROI 5), and less solute spreads towards the posterior ventricle (ROI 6).

### Modification of ventricular geometry impacts solute distribution

**Fig. 6:**
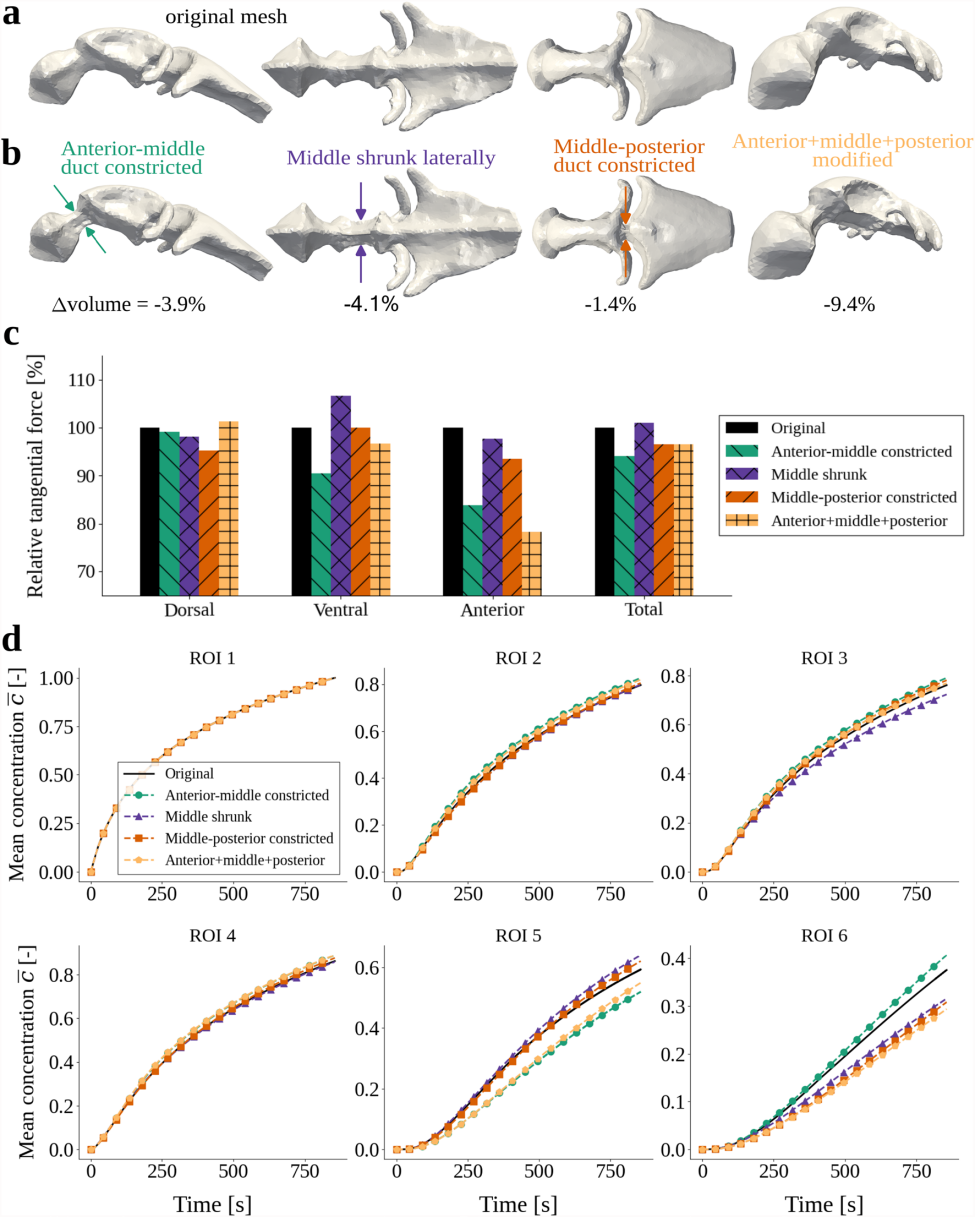
**a** The original computational mesh of the ventricular geometry. **b** The four modified versions of the computational geometry. Compared to the original mesh, the volumes of the deformed meshes are reduced by 3.9%, 4.1%, 1.4% and 9.4%. **c** Mean concentration $\overline{c}(t)$ simulated with the original and modified geometries in the six regions of interest (ROIs). **d** A comparison of the tangential cilia force in the three cilia regions and the total force applied, calculated with Equation (6). In each region, the force $F_{\text{original}}$ applied on the original mesh is the baseline of 100%, to which the forces $F_i$ of the other mesh versions are compared, where *i* denotes the modification scenario. The bars for the four alternative scenarios display $F_i/F_{\text{original}}\times 100\%.$

Variations in ventricular morphology impact CSF flow patterns, and we previously identified large interindividual variation in ventricular size and CSF velocities between embryos at given developmental stages and throughout development [14, 18, 58]. To assess how these variations influence solute distribution, we consider four alternative ventricular geometries: (i) constriction of the anterior-middle interventricular duct (66 % reduction in cross-sectional area); (ii) constriction of the middle-posterior duct (33 % reduction); (iii) middle ventricle shrunk in the lateral direction (43 % reduction); and (iv) all three combined (Fig. 6a, b). In these four modified geometries, the total volume is reduced by 3.9%, 4.1%, 1.4% and 9.4%, respectively, compared to the original geometry. As a result of the geometry modifications, the forces applied with the cilia tangential traction differ across the scenarios (Fig. 6c).

Constricting the anterior-middle duct reduces solute transport to the anterior ventricle (ROI 5), and the mean concentrations in ROIs 2–4 and 6 slightly increase, compared to the original geometry (Fig. 6d). Constricting the middle-posterior duct mainly delays the transport into the posterior ventricle (ROI 6), while slightly accelerating transport towards the anterior ventricle (ROI 5). Thus for both scenarios, solute transport is restricted through the respective constricted interventricular duct. For the geometry with a shrunk middle ventricle, we observe faster transport to the anterior ventricle (ROI 5) and slower transport to the lower middle and posterior ventricles (ROI 3, ROI 6). Interestingly, this reduction in the transport to the posterior ventricle (ROI 6) happens in spite of a 6.6% increase in the ventral cilia forces, which are the cilia that propel CSF towards the posterior ventricle.

When combining all three modifications, we observe higher retention of solute in the middle ventricle (ROIs 2–4), while the mean concentrations are reduced in both the anterior and the posterior ventricles (ROIs 5 and 6). To summarize, these results show that the ventricular geometry and the cross-sectional area of the ventricular ducts clearly impact solute transport, with notable differences in transport resulting from modest geometry modifications. These findings may also explain the larger differences in the cilia-deficient computational model results compared to the differences in experimental data in Fig. 4, as the computational geometry is not an exact representation of the individual zebrafish ventricles.

## Discussion

In this study, we have presented a computational model of CSF flow and solute transport within brain ventricles. We used the embryonic zebrafish brain ventricles since their geometry and flow mechanisms are well described, thereby allowing for validation of the simulation results against experimental measurements. Our computational framework can be adapted to other ventricular systems, once their flow parameters are adequately resolved. Here, we assumed that intraventricular CSF flow was governed by the Stokes equations. By imposing tangential traction and normal pressure acting on the CSF, we were able to reproduce flow features observed experimentally. In particular, vortex-structured flow mediated by the cilia and pulsatile flow mediated by the cardiac cycle emerged as key flow characteristics.

Using the simulated CSF flow, we modeled solute transport via an advection–diffusion equation. The resulting evolution and distribution of solutes compared well with data from in vivo experiments in zebrafish and is advection-dominated on the global scale for a physiologically-relevant range of solutes. By studying the distribution patterns in the absence of cilia motion, both via zebrafish mutants and computationally, we find that diffusion more strongly affects solute spread to distant parts of the ventricular system (especially upstream). Regional loss of cilia delayed solute transport with the most pronounced effect stemming from dorsal cilia paralysis. Moreover, the ventricular morphology also affects solute distribution, with the duct between the anterior and middle ventricles as a key passage. Altogether, we present not only new findings on the roles of advection and diffusion in brain ventricular transport, but also provide a computational framework to study CSF flow and transport in ventricular geometries.

### The roles of advection and diffusion in brain ventricular solute transport

We quantified brain ventricular solute transport based on changes in fluorescence intensity $\Delta F$ (experiments) and mean concentration $\overline{c}$ (simulations) over time. Our results show that the solute distribution depends on a balance between advection and diffusion. While advection is the strongest transport mechanism on a global scale for the three solutes we studied (global Péclet numbers $> 1$), diffusion is balancing out advection for shorter distances and in regions without motile cilia activity. For instance, for the larger extracellular vesicles, no solute reached the distant posterior ventricle (a distance of $L = 200$ µm) within the simulation time (around 860 s). Advective transport by the mean velocity $\overline{U}=2.4$ µm/s over the same distance would occur on a timescale of $t_a=L/\overline{U}=83$ s, indicating that transport to the posterior ventricle is not driven by advection at this developmental stage. The estimated timescale of diffusion for the extracellular vesicles ($t_d=L^2/D_1$) over the same distance is 24500 s, but as low as 696 s and 348 s for the smaller proteins STM-GFP and Dendra2, respectively. These estimates support our observations that STM-GFP and Dendra2 spread to the posterior ventricle (by diffusion) while the extracellular vesicles do not. Larger molecules transported primarily by advection are thus more easily compartmentalized within the ventricles as a result of the compartmentalized CSF flow.

### Effects of cilia motility on transport

Removing motile cilia delayed transport within the middle ventricle and towards the anterior ventricle, while the transport to the posterior ventricle was unaffected. Photoconversion experiments with cilia mutant zebrafish embryos (*smh*) aligned with these observations. These simulations and experiments further indicate that transport from the middle to the posterior ventricle is purely diffusive at this developmental stage, while transport within the middle ventricle depends on cilia motility. Furthermore, our experimental results provide quantification of the impact of cilia motility on brain ventricular transport. Interestingly, Yoshida et al. observed a drastic reduction in CSF mixing in specific regions of the human brain ventricles when simulating reduced cilia motility [52], an observation that compares well with our findings. We observed a clear fluid flow compartmentalization induced by ciliary motion in our experimental system, however it remains to be identified whether the vorticity structure has a biological significance.

### Model validation

Our computational predictions and in vivo experimental measurements agree to a large extent. Examining any discrepancies in more detail indicates that the simulated transport dynamics are more rapid and results in higher total amounts of solute. Differences between experiments and simulations may relate to ventricular size, as we showed that modifying the ventricular geometry impacts solute distribution, and the ventricular size varies substantially in the control population at 2 dpf. In addition, differences may result from the definition of the photoconversion site, since the volume of this region determines the total amount of solute and thereby the rate of transport. Another source of discrepancy may relate to the permeability of the ventricular wall, which we considered as impermeable in our simulations. The neuroepithelium lining the walls of brain ventricles in 24 h old zebrafish is permeable to dyes smaller than 70 kDa [68]. If this is also the case at 2 dpf, Dendra2 (which are 26 kDa in size [69]) would leak out of the brain ventricles over the relevant time spans and render the dynamics slower. It may also be the case that our model predicts faster transport because the Dendra2 diffusion coefficient we used (based on an experimental value [70]) is too high. This could be because embryonic CSF has higher viscosity than the solution considered in the measurements, or because the presence of proteins, nutrients and other solutes obstructs diffusive transport [71–73]. Finally, an important difference is the three-dimensional nature of the computational model, while the experimental images only show 2D slices.

We modeled motile cilia as a tangential traction at the ventricular walls while allowing the CSF to slip, motivated by experimental observations of flow fields in embryonic zebrafish brain ventricles in which the fluid velocity increases towards the wall (Fig. 1c) [14]. The ciliated boundaries in our model are regionally homogeneous, whereas in reality cilia populate the ventricle walls heterogeneously [14]. Heterogeneity in cilia location, beating direction and beating frequency enhances particle clearance in airway cilia arrays in mice [54]. Beating directionality can affect transport, and specific coordination of the ciliary beating is important for flow and signaling processes in the brain ventricles [14, 24, 33, 42, 55, 61, 62], for physiological mucous transport in airways [26, 74–76], and in the nodal flow that establishes left-right symmetry in mammals [63, 64]. Incorporating spatial heterogeneity in our model would therefore presumably affect results, and could be interesting to pursue in further work.

The maximum stress of the cilia boundary condition was $\vert \varvec{\tau }\vert _{\text{max}} = 2.5 \ \text{mPa} = 2.5 \times 10^{-3} \ \mathrm {N/m}^2$. In a model of CSF flow in human brain ventricles, Siyahhan et al. modeled ciliary motion with a force increasing linearly from the ventricular wall to a maximum force density of $f_{\text{max}} = 526 \ \mathrm {N/m^3}$ at a distance 15 µm (the cilium length) away [32]. Their simulation results suggest that cilia only contribute to near-wall flow dynamics, which contrasts our results in which the cilia induce large-scale flow. This is a consequence of the scale of the cilia and the difference in ventricular size between humans and zebrafish. In a cilia array of thickness $\delta = 15$ µm, the maximum stress in our model would correspond to a maximum force density of

$$\begin{aligned} f_c = \frac{\vert \varvec{\tau }\vert _{\text{max}}}{\delta } = \frac{2.5 \times 10^{-3} \ \mathrm {N/m}^2}{15 \times 10^{-6} \ \text{m}} = 169 \ \mathrm {N/m^3}. \end{aligned}$$

With $\delta =5$ µm (the typical cilium length in zebrafish brain ventricles [14, 30, 53]) our cilia stress corresponds to a maximum force density of $f_c=507 \ \mathrm {N/m^3}$, similar to the value Siyahhan et al. used [32]. For further comparison, Thouvenin et al. modeled two-dimensional cilia-mediated flow in the central canal of zebrafish embryos using a force density of 4000 $\mathrm {N/m^2}$[29]. Averaging this value over the reported central canal diameter 8.9 µm yields a maximum force of 449 $\mathrm {N/m^3}$. In light of the previous finding that motile cilia align by response to shear stress magnitudes of flow, [24] it is interesting to note the above similarity in cilia force estimates, despite the variety in application.

### Model limitations

Since we focused our study on the effect of motile cilia, we neglected physiological effects other than cilia and cardiac pulsations on ventricular flow, which include among others bodily movement, CSF production and clearance, and interstitial fluid dynamics. The approach used to model cardiac pulsations is simplified for several reasons. One is due to a lack of knowledge in the processes driving cardiac pulsations of CSF, which may relate to blood vessel contraction and dilatation, or displacement of the ventricular wall and brain parenchyma. The description of these physiological processes in embryonic zebrafish brain ventricles is not as extensive as that for the human brain. We therefore neglected wall displacements, blood vessel movement and fluid displacement across the ventricular walls. Second, our study considers a younger brain that does not have a fully developed ventricular, cardiovascular and nervous system. Including these parameters would however be highly relevant to model a more developed zebrafish, where we expect that the above mentioned physiological effects will be more prominent. Third, availability of particle tracking data allowed for calibrating our cardiac pulsations model by comparing numerical and experimental CSF velocities, since there is only one parameter, the normal pressure amplitude, that needs to be determined.

The simplified approach to modeling cardiac pulsations resulted in CSF flow in and out of the anterior/posterior ends of the ventricles. When simulating solute transport, we handled the advective transport through these boundaries via an approximate periodic boundary condition. This condition lets solute exit at the anterior or posterior boundary and re-enter at the other side to conserve the total solute concentration, to reflect our assumption that the embryonic zebrafish ventricular system is closed at 2 dpf. This boundary condition may influence the second-order accuracy of the advection–diffusion equation discretization. To ensure that the model implementation did not introduce excessive numerical diffusion, we performed a refinement study of the transport model (see Appendix B.4). A possible improvement of the approximate periodic boundary condition would be to consider a 3D-0D coupling of the in- and outflow using ordinary differential equations, similar to applications in cardiac modeling [77, 78]. Alternatively, the beating cardiac motion could be modeled by ventricular wall deformations to avoid advective boundary fluxes [13, 79–81], in the case that a quantitative description of such wall deformations for the embryonic zebrafish brain ventricles were present.

The Brezzi-Douglas-Marini and discontinuous Galerkin scheme $\text{BDM}_1$-$\text{DG}_0$ used to discretize the Stokes equations is a first-order approximation. Because of consistent results under a refinement study (see Appendix B.3) as well as qualitative replication of experimentally observed flow fields, we deemed the approximation satisfactory for the purposes of this study. We also remark that the $\text{BDM}_1$-$\text{DG}_0$ scheme is computationally more expensive than more common discretizations such as Taylor-Hood ($P_2-P_{1}$ elements) [82] or lower-order enriched elements (e.g. MINI) [83]. However, we prefer the BDM-DG scheme because of its mass conservation properties: the numerical approximation of the CSF velocity $\varvec{u}_h$ satisfies the continuity equation (Equation (1b)) point-wise, an important property for the stability of the advection–diffusion equation, especially in advection-dominated cases [84–86]. Additionally, the BDM-DG scheme lets us impose the impermeability condition $\varvec{u}\cdot \varvec{n}=0$ directly [87] (see Appendix A.1 for more details).

### Outlook

Altogether, we have shown that the flow generated by ciliary motion in embryonic zebrafish brain ventricles is of the scale of the geometry [14]. In contrast, one can expect that in large brains such as the human brain, ciliary beating contributes only to flow proximal to the ventricular walls [32]. Nevertheless, the cilia may still play an important role in regulating the amount of substances, especially extracellular vesicles, close to the ventricular walls, similar to coral reefs where cilia are primary regulators of oxygen amounts at the coral surface [20]. Notably, the discovery of a cilia-driven flow network in rodent brains strongly supports such selective brain ventricular transport [33]. Our results also revealed that solute concentrations are affected by ventricular geometry changes. In fact, a 9% difference in volume had a noticeable impact on solute transport, suggesting that pathological ventricular volume changes, such as the enlargement observed in hydrocephalus, could affect CSF distribution and composition. Because CSF plays an important role in the regulation of solutes within the ventricular system [88], understanding more of the interplay between transport dynamics and ventricular morphology, as well as the impact of reduced mixing resulting from cilia-deficiency, are important considerations for future work.

## Methods

### Zebrafish maintenance, genotyping and strains in photoconversion experiments

The animal facilities for zebrafish (*danio rerio*) are approved by the Norwegian Food Safety Authority (NFSA, Mattilsynet). The zebrafish were maintained in accordance with the guidelines set by the NFSA and the European Communities Council Directive.

The embryonic and adult zebrafish were raised under standard husbandry conditions at $28.5^{\circ }$C in a Techniplast Zebtech Multilinking system. The fish tanks were kept at constant pH 7.0 and 685 µS with a 14/10 hr light/dark cycle. From fertilization to 3 days post-fertilization (dpf), embryos were maintained in egg water (1.2 g marine salt and 0.1% methylene blue in 20 L reverse osmosis (RO) water) and subsequently transferred to artificial fish water (AFW) (1.2 g marine salt in 20 L RO water). The zebrafish lines used in the experiments were *ccdc103(dnaaf19)*^*tn222a*^ (*schmalhans (smh)*) [89] and *Tg(gfap:Gal4FF)*^*nw7Tg*^ [90], *Tg(5xUAS:Signal-Dendra2)*^*nw21Tg*^. Animals were in the pigmentless *nacre*^*b692*^ (*mitfa*^-/-^) [91] background.

Wholemount in vivo live imaging and photoconversion experiments were performed with zebrafish embryos at the 2 dpf stage obtained from inbreeding of heterozygous *smh*^±^*;Tg(gfap:Gal4FF);Tg(5xUAS:Signal-Dendra2*) adult animals, as described by D’Gama et al. [18]. Controls consisted of either wild-type (*smh*^+/+^) or *smh*^±^ heterozygous zebrafish from the same breeding. The mutants were identified based on their curved body [89].

For genotyping, genomic DNA (gDNA) was isolated from clipped fins of anesthetized adult fish using 100 µL of PCR lysis buffer (containing 1 M tris pH 7–9, 0.5 M EDTA, tritonX-100, and Proteinase K 0.1 mg/ml) overnight at $50^{\circ }$C. To stop the lysis reaction, the samples were heated to $95^{\circ }$C for 10 min and then centrifuged at 13000 rpm for 2 min. The supernatant containing gDNA was utilized for KASP assays-based analysis. The gDNAs were diluted (1:2) with water. The KASP assay was run following the guidelines of the manufacturer (LGC Biosearch Technologies^TM^) with 3 µL gDNA, 5 µL of master mix, 0.14 µL of assay mix and 1.86 µL of RO water per well on a 96-well plate.

### Generation of transgenic line to study CSF and transport dynamics

To generate a transgenic line expressing the photoconvertible protein Dendra2 in the brain ventricles, we fused the open reading frame (ORF) of the neuropeptide y (npy) signal peptide (*npy*: ENSDARG00000036222, Q1LW93) to the N-terminal of zebrafish codon optimized Dendra2 DNA sequence. The DNA sequence was synthesized with EcoR1 enzyme sites at the 5’ and 3’ ends (GenScript Biotech) and inserted into the pT2MUASMCS vector [92] through restriction enzyme cloning (GenScript Biotech) to generate the 5xUAS:Signal-Dendra2 plasmid DNA.

A volume of 2 nl of a mixture of the plasmid DNA (60 pg) and tol2 mRNA (10 pg) was microinjected into one-cell stage embryos to generate the transgenic line, as described in Jeong et al. [58]. The injected embryos were raised to adulthood (F0). Germline-transmitted founder zebrafish were identified by breeding with multiple Gal4 transgenic lines. Stable F1 embryos expressing the Gal4-driven Dendra2 signals were screened and raised to adult zebrafish.

### Wholemount zebrafish in vivo time-lapse imaging and photoconversion of Dendra2

Two dpf embryonic zebrafish were anesthetized in 0.013% MS222 (Ethyl 3-aminobenzoate methanesulfonate, Sigma) in AFW and mounted laterally in 1.5% low melting agarose in a Flurodish (VWR, FD35PDL-100), to obtain a lateral view of the brain ventricles. After the fish were positioned in the agarose, the dish stood for 5 min to allow the agarose to solidify. After solidification, AFW containing 0.013% MS222 was added, and then the fish were transferred to the confocal microscope (LSM880 Examiner, Zeiss) and imaged using a 20x water-immersion objective (Zeiss, NA 1.0, Plan-Apochromat) at room temperature.

The time-lapse images were acquired in a single plane, covering the telencephalic, diencephalic and rhombencephalic ventricles simultaneously, with a frequency of 0.37-$-$0.38 Hz (2.60-$-$2.68 sec / frame). The size of the images was $1024\times 400$ pixels. In total, 300 images were acquired per fish. The first 10 images were scanned without photoconversion to obtain a baseline value of fluorescence intensity, and the next 290 images were scanned while performing photoconversion.

For photoconversion, “Bleaching" and “Region" in the ZEN software were used. A 405 nm laser was focused into a circular area (diameter: 16 µm, scan 0.77 µsec/pixel) of the dorsoposterior diencephalic ventricle with 100% laser power. The laser was illuminated in the area repeatedly between each scanning. After imaging, the fish health was checked and only data from healthy fish were analyzed. Data were collected and analyzed from three separate experiments, with a total of 8 controls and 6 mutants.

### Photoconversion data analysis

The image data analysis was performed in MATLAB and the codes are openly available [93]. The photoconverted channel was aligned to correct for drift in *x*, *y* directions using previously developed codes [28, 30]. Only stable recordings were further analyzed. To increase the signal-to-noise ratio, the images were downsampled by a block average using a resampling factor of 6. The change in fluorescence intensity $\Delta F = (F-F_0)/F_0$ was calculated for each pixel and each time point. Here, the value $F_0$ is the average fluorescence intensity for the baseline (the first 10 time-lapse images) acquired before photoconversion.

To identify the time needed to pass a certain threshold value, $\Delta F$ values were first smoothened. The first timepoint when values surpassed the threshold was reported per pixel (in the downsampled images). A mask for the image was generated using the green (not photoconverted) channel and calculated on the block-averaged data based on the intensity being higher than 1.5x median intensity of all the frames.

To obtain $\Delta F$ values for each region of interest (ROI), ROIs were first drawn on the aligned and block-averaged time series (Fig. 2). Six ROIs were drawn manually: ROI 1 around the location of photoconversion, and ROIs 2–6 in different regions of the ventricular system. The pixel values of the fluorescence intensity within one ROI were averaged for each time point, and the relative change $\Delta F$ calculated with this averaged value. To normalize the data with respect to photoconversion efficiency, the $\Delta F$ values for all ROIs were divided by the $\Delta F$ values obtained for ROI 1 at the end of the experiment, so that $\Delta F$ values for the photoconverted site ROI 1 ranged from zero to one. Finally, we averaged the $\Delta F$ data from controls and from mutants.

### Intraventricular injection of microbeads and particle tracking

We carried out particle tracking in an embryonic zebrafish injected with fluorescent beads, as described in previous work [14]. Anesthetized 2 dpf zebrafish were injected with 1 nl of a mixture containing 0.1% w/v fluorescent beads (SPHERO Fluorescent Yellow Particles 1% w/v, F = 0.16 mm) diluted in 7.5 mg/ml 70 kDa rhodamine B isothiocyanate-dextran (RITCdextran; Sigma-Aldrich, R9379) dissolved in artificial CSF. Artificial CSF composition was as follows: 124 mM NaCl, 22 mM D-(+)-Glucose, 2.0 mM KCl, 1.6 mM MgSO_4_ $\cdot$ 7 H_2_O, 1.3 mM KH_2_PO_4_, 24 mM NaHCO_3_, 2.0 mM CaCl_2_ $\cdot$ 2 H_2_O. The needles used for the injections were pulled with a Sutter Instrument Co. Model P-2000 from thin-walled glass capillaries (1.00 mm; VWR) and cut open with a forceps. A volume of 1 nl of the solution was injected with a pressure injector (Eppendorf Femtojet 4i) in the rostral rhombencephalic ventricle. The pressure and time were calibrated for each needle using a 0.01 mm calibration slide.

Following injection, the zebrafish was directly placed under the confocal microscope and 1200 images were acquired at a frequency of 13.1 Hz. A single optical section was obtained. Particle tracking was performed using TrackMate [94] in Fiji/ImageJ [95] and plotted using MATLAB, as described by Jeong et al. [40]. The parameters for TrackMate were as follows. The DoG detector was used with the settings: threshold: 2.0, with median filtering; radius: 2.0, with subpixel localization. Next, the Simple LAP tracker was used with settings: max frame gap: 2; alternative linking cost factor: 1.05; linking max distance: 5.0; gap closing max distance: 2.0; splitting max distance: 15.0; allow gap closing: true; allow track splitting: false; allow track merging: false; merging max distance: 15.0; cutoff percentile: 0.9. Only the particle tracks with at least 30 data points, while simultaneously covering a distance of at least 8.8 µm, are shown (Fig. 1b, c).

### Image-based computational geometries of zebrafish brain ventricles

To model CSF flow and transport in the zebrafish brain ventricles, we generated a 3D surface representation of the ventricular walls using confocal imaging data of a zebrafish embryo injected intraventricularly with a 70 kDa dye at 2 dpf [14]. At 2 dpf, the ventricular system consists mainly of 3 cavities: the telencephalic (anterior), the di-/mesencephalic (middle) and the rhombencephalic (posterior) ventricles, which are connected by interventricular ducts. From the surface geometry, we constructed a volumetric mesh of the ventricles by initially using fTetWild [96] and subsequently local refinement functionality in DOLFINx [97]. The geometry spans approximately 600 microns along the rostrocaudal axis and around 300 microns in the lateral direction. At most around 100 microns separates the ventral and dorsal ventricular walls. The standard mesh consists of 141 512 tetrahedral cells with a maximal (minimal) edge length of 14.9 µm (3.1 µm) (Fig. 1d). Parts of the ventricular surfaces were marked as lined with motile cilia (Fig. 1d, magenta markers) [14]. The surface geometry from confocal imaging data and all computational meshes used in this work are openly available [93].

The morphology and geometry of the brain ventricles vary across zebrafish individuals, and under physiological and pathological conditions [14]. Motivated by an interest in how ventricular geometry impacts solute distribution, we also consider four variations in the geometry (cross-sectional area reduction in parentheses): by shrinking the fore-mid brain connection (66 %), the middle ventricle (33 %), the mid-hind brain connection (43 %), and all three simultaneously. We used Blender [98] to modify the geometries (Fig. 6a, b).

### Computational CSF dynamics in the brain ventricles

The beating motion of the cilia generates CSF flow with persistent rotational structures [14] within the brain ventricles (Fig. 1a–c). In addition, cardiac pulsations induce pulsatile CSF flow. We model this flow of CSF by the time-dependent, incompressible Stokes equations which read as follows. Find the CSF velocity $\varvec{u}= \varvec{u}(\varvec{x}, t) = (u_x(\varvec{x}, t), u_y(\varvec{x}, t), u_z(\varvec{x}, t))$ and the CSF pressure $p = p(\varvec{x}, t)$ for $\varvec{x}\in \Omega$ and time $t\in (0, T]$, such that

1a $$\begin{aligned} \rho \frac{\partial \varvec{u}}{\partial t} - \nabla \cdot \boldsymbol{\sigma }&= \textbf{0} \quad \text {in } \Omega \times (0, T], \end{aligned}$$

1b $$\begin{aligned} \nabla \cdot \varvec{u}&= 0 \quad \text {in } \Omega \times (0, T], \end{aligned}$$

where the stress tensor is defined as $\boldsymbol{\sigma }(\varvec{u}, p) = 2\mu \boldsymbol{\varepsilon }(\varvec{u}) - p\textbf{I}$ and $\boldsymbol{\varepsilon }(\varvec{u}) = \frac{1}{2}\left( \nabla \varvec{u}+ (\nabla \varvec{u})^T\right)$ is the strain-rate tensor. Bold-face characters denote vectors or tensors, and $\textbf{I}$ is the identity tensor in three dimensions. Furthermore, $\rho =1000 \ \mathrm {kg/m^3}$ is the CSF density and $\mu =0.7 \ \mathrm {mPa\cdot s}$ is the dynamic viscosity of the CSF [71]. We introduce the tangential traction $\hat{\boldsymbol{\sigma }}_{\parallel }$ as the tangential component of the traction $\hat{\boldsymbol{\sigma }}=\boldsymbol{\sigma }\varvec{n}$:

$$\begin{aligned} \hat{\boldsymbol{\sigma }}_{\parallel }= P_{\varvec{n}}(\hat{\boldsymbol{\sigma }}) = (\textbf{I} - \varvec{n}\otimes \varvec{n})\hat{\boldsymbol{\sigma }}, \end{aligned}$$

where $\varvec{n}$ is the outer unit normal of the boundary surface $\Gamma = \partial \Omega$, and $P_{\varvec{n}}(\varvec{r})$ is the tangential projection of a vector $\varvec{r}$ onto $\Gamma$. We consider three types of boundary conditions: (i) *tangential traction* (no flow normal to the boundary $\varvec{u}\cdot \varvec{n}= 0$ with a stress $\varvec{\tau }$ applied tangentially on the boundary $\hat{\boldsymbol{\sigma }}_{\parallel }= \varvec{\tau }$), (ii) *normal pressure* (a pressure $\tilde{p}$ applied in the normal direction: $(\mu \nabla \varvec{u}- p\textbf{I})\varvec{n}= \tilde{p}(t)\varvec{n}$), and (iii) *free slip* (no flow normal to the boundary $\varvec{u}\cdot \varvec{n}= 0$ and no tangential traction $\hat{\boldsymbol{\sigma }}_{\parallel }= \textbf{0}$). We assume that the system starts at rest: $\varvec{u}(\varvec{x}, t=0) = \textbf{0}$.

To study the impact of the motile cilia and the cardiac pulsations on the CSF flow, we consider the following computational flow scenarios, each predicting a CSF flow velocity $\varvec{u}$ and pressure *p*. In all of the scenarios, $\Gamma _{\text{s}}$ denotes a free-slip boundary.

- A *baseline* flow model representing a wild-type zebrafish including contributions both from the cilia forces and the cardiac pulsations. In this scenario, we let $\Gamma =\Gamma _{\text{s}}\cup \Gamma _{\text{c}}\cup \Gamma _{\text{p}}$ and apply the tangential traction $\varvec{\tau }$ on the cilia boundary $\Gamma _{\text{c}}$ and prescribe the normal pressure $\tilde{p}$ on the anterior/posterior boundary $\Gamma _{\text{p}}$.
- A *cilia-driven/no-cardiac* flow model including contributions from the cilia forces but no cardiac pulsations. Here, $\Gamma =\Gamma _{\text{s}}\cup \Gamma _{\text{c}}$, and we apply a tangential traction $\varvec{\tau }$ on the cilia boundary $\Gamma _{\text{c}}$. In the absence of cardiac-induced flow, the anterior/posterior boundary $\Gamma _{\text{p}}=\emptyset$.
- A *cardiac-induced/no-cilia* flow model including contributions from the cardiac pulsations but no cilia forces. In this scenario, we disregard the cilia and consider $\Gamma =\Gamma _{\text{s}}\cup \Gamma _{\text{p}}$, prescribing the normal pressure $\tilde{p}$ on the anterior/posterior boundary $\Gamma _{\text{p}}$.

The tangential traction and normal pressure are described further below.

#### Cilia-driven flow

On the ciliated regions of the ventricle walls $\Gamma _{\text{c}}$ (Fig. 1d, magenta markers), we impose a constant-in-time tangential traction $\hat{\boldsymbol{\sigma }}_{\parallel }=\varvec{\tau }$ to represent the net forces of the cilia acting on the CSF. We set $\varvec{\tau }(\varvec{x}, \varvec{r}) = \tau \lambda (\varvec{x}) P_{\varvec{n}}(\varvec{r})$, where the function $\lambda (\varvec{x})$ and the sign of the vector $\varvec{r}=\pm (1, 0, 1)$ varies depending on the cilia region (see Appendix A.1). Previous experiments [14] indicate flow speeds proximate to the ventricular walls of $27.4 \pm 5.4$ µm/s and $2.6 \pm 0.6$ µm/s in the dorsal and ventral regions of the middle ventricle, respectively. In the anterior ventricle, the speeds were estimated at $4.7 \pm 1.3$ µm/s. We calibrated $\varvec{r}$ and $\tau =0.65$ mPa by comparison of the simulated maximum velocity magnitude with these flow speeds.

#### Flow induced by cardiac pulsations

To generate a cardiac-induced pulsatile flow inside the brain ventricles, we set $\tilde{p}(t)=0$ and $\tilde{p}(t)=-A\sin {\omega t}$ on anterior and posterior parts, respectively, of the boundary $\Gamma _{\text{p}}$ (Fig. 1d, blue markers). Based on a measured cardiac frequency of $f=2.22$ Hz [14], we set the angular cardiac frequency $\omega =2\pi f=6.97$ rad/s. The amplitude $A=1.5$ mPa determines the magnitude of the pulsatile flow and was calibrated based on previous experiments [14]. Particle tracking data was used to determine the contributions of the cardiac pulsations to the velocity magnitude, as compared to the effect of cilia motion on the flow (cf. Appendix C).

### Computational model of solute transport after photoconversion

We simulate transport of photoconverted proteins within the brain ventricles by modeling transport of a solute with concentration $c(\varvec{x}, t)$ for $\varvec{x}\in \Omega$ and time $t\in (0, T]$ by the advection–diffusion equation

2 $$\begin{aligned} \frac{\partial c}{\partial t} + \nabla \cdot \varvec{J}= 0 \quad \text {in } \Omega \times (0, T], \end{aligned}$$

where the total concentration flux $\varvec{J}$ is assumed to consist of an advective and a diffusive flux:

$$\begin{aligned} \varvec{J}= c\varvec{u}- D\nabla c . \end{aligned}$$

Here, $\varvec{u}$ is the CSF velocity field, governed by the Stokes equations (Equation (1)), and *D* is the molecular diffusion coefficient of the solute. The ventricles are assumed to be a closed system, with impermeable ventricular walls. Therefore, we impose a no-flux condition $\varvec{J}\cdot \varvec{n}=0$ at the boundary $\Gamma \setminus \Gamma _{\text{p}}$. At the anterior/posterior boundary $\Gamma _{\text{p}}$, solute concentration may be advected in and out of the domain by the CSF flow. To approximately achieve conservation of the total solute concentration, we impose the following boundary conditions on $\Gamma _{\text{p}}$. On the outflow part of $\Gamma _{\text{p}}$ ($\varvec{u}\cdot \varvec{n}\ge 0$), we impose zero diffusive flux ($D\nabla c\cdot \varvec{n}= 0$). The total flux on the inflow part of $\Gamma _{\text{p}}$ ($\varvec{u}\cdot \varvec{n}< 0$) is set equal to the total outflow flux at the previous timestep to approximate a periodic boundary condition. See Appendix A.3 for further details.

To computationally represent protein photoconversion in a region $\Omega _{\text{pc}} \subset \Omega$ (corresponding to ROI 1), we prescribe a given time-dependent value to the solute concentration in this region. Mimicking the experimental protocol, we consider the dorsal part of the diencephalic ventricle as the subdomain $\Omega _{\text{pc}}$, and set

3 $$\begin{aligned} c(\varvec{x}, t)=\frac{\log {(1+t/a)}}{\log {(1+T/a)}}, \quad \varvec{x}\in \Omega _{\text{pc}}, \quad t\in (0, T]. \end{aligned}$$

The value $a=65$ was chosen to fit the fluorescence intensity curve observed in the physical photoconversion experiments. Because steep gradients in the concentration field may arise from imposing $c(\varvec{x}, t)$ in $\Omega _{\text{pc}}$, the mesh is locally refined around this region.

In order to impose a smooth initial condition, we initially solve the diffusion equation

$$\begin{aligned} \frac{\partial c}{\partial t}&= \tilde{D}\nabla ^2 c &&\text {in } \Omega \times (0, 2\Delta t]\\ c(\varvec{x}, t=0)&= 0 &&\text {in } \Omega \end{aligned}$$

with an increased diffusion coefficient $\tilde{D}=D\times 10^{5}$ for two timesteps. The solutions are normalized by their respective maximum values, and subsequently multiplied with the value of Equation (3) at the times $t=\Delta t$ and $t=2\Delta t$, respectively. The resulting concentration fields are used as initial conditions for $c(\varvec{x}, 0)$ and $c(\varvec{x}, \Delta t)$ for all $\varvec{x}\in \Omega$, where $\Delta t$ is the timestep. The two initial conditions are required because of the two-step temporal discretization scheme employed (see "Numerical approximation of the advection-diffusion equation" section for more details.)

### Estimation of diffusion coefficients via the Stokes-Einstein relation

Motivated by an interest in molecules that are significant in neural development, we study the transport of molecules resembling (i) the Dendra2 Fluorescent Protein (Dendra2) used in the photoconversion, (ii) the Starmaker+Green Fluorescent Protein (STM-GFP) reported by Jeong et al. [58], and (iii) extracellular vesicles. To estimate their diffusion coefficients, when not available in the literature, we use the Stokes–Einstein relation

4 $$\begin{aligned} D = \frac{k_B T}{6\pi \mu R}, \end{aligned}$$

which is an expression for the diffusion coefficient of a spherical molecule suspended in a viscous fluid [72]. In Equation (4), $k_B = 1.38 \times 10^{-23}$ J/K is the Boltzmann constant, $T = 310$ K is the absolute temperature, $\mu$ is the dynamic viscosity of the CSF, and *R* is the radius of the molecule. For the extracellular vesicles, we calculate *D* using Equation (4) with the relatively large $R=150$ nm [99]. For STM-GFP, we estimate the diffusion coefficient by extrapolating the experimentally observed diffusion coefficient of GFP [100, 101], assuming that if *D* scales with 1/*R* according to Equation (4), *D* would scale with the cube root of the molecule mass [102]. The resulting diffusion coefficients are $D_1$ (extracellular vesicles), $D_2$ (STM-GFP) and $D_3$ (Dendra2) as given by Table 1.

**Table 1:** Molecular mass and diffusion coefficients of simulated solutes

Molecule	Label	Mass [kDa]	$D \ [\mathrm {m^2/s}]$	References
Extracellular vesicles (radius of 150 nm)	$D_1$	–	$2.17 \times 10^{-12}$	†
GFP (saline aqueous solution)	–	26.9 [103]	$8.70 \times 10^{-11}$	[100, 101]
STM-GFP	$D_2$	66.2 [104] + 26.9 [103]	$5.75 \times 10^{-11}$	*
Dendra2 Fluorescent Protein	$D_3$	25.6 [69]	$1.15 \times 10^{-10}$	[70]

STM and GFP denote Starmaker Protein and Green Fluorescent Protein, respectively. (*) The value was extrapolated based on the value of *D* for GFP reported in the literature [100, 101], assuming that *D* scales with the cube root of the molecule mass. (†) The value was estimated using the Stokes-Einstein relation Equation (4)

### Quantities of interest

We calculate the mean solute concentrations

5 $$\begin{aligned} \overline{c}_i(t) = \frac{1}{|\Omega _i|}\int _{\Omega _i} c(\varvec{x}, t)\,\, \text{d}\varvec{x}, \end{aligned}$$

in the six regions of interest (ROIs) $\Omega _i$ ($i = 1, \dots , 6$) as functions of time, where $|\Omega _i|$ is the volume of the domain $\Omega _i$. The dynamics of ROI 1 follows directly from Equation (3). In addition, we report the time-to-threshold as the time $\hat{t}_i$ when the mean concentration $\overline{c}_i$ first exceeds a threshold value $\hat{c}_i$. We use $\hat{c}_i=0.25$ for ROI 1–4 and $\hat{c}_i=0.10$ for ROI 5–6.

To evaluate the relative importance of advection and diffusion as transport mechanisms, we consider the Péclet number

$$\begin{aligned} \text{Pe} = \frac{\mathrm {diffusion \ timescale}}{\mathrm {advection \ timescale}} = \frac{t_d}{t_a} = \frac{L_p^2/D}{L_p/U_p} = \frac{L_p U_p}{D}. \end{aligned}$$

Thus, if $\text{Pe} > 1$, transport is dominated by advection, and if $\text{Pe} < 1$ transport is dominated by diffusion. Here, $L_p$ and $U_p$ are characteristic length and velocity scales, respectively, and *D* is the solute diffusion coefficient. In calculating a global Péclet number, we use $L_p=600$ µm, the approximate length of the ventricular geometry along the rostrocaudal (*x*-) axis. We use a mean velocity $\overline{U}$ as the characteristic velocity $U_p$. The mean velocity $\overline{U}$ is calculated by first averaging the velocity in space:

$$\begin{aligned} \overline{u}^2 = \frac{1}{|\Omega |}\int _{\Omega }\varvec{u}\cdot \varvec{u}\, \text{d}\varvec{x}, \end{aligned}$$

and then averaging $\overline{u}$ in time over one cardiac cycle to yield $\overline{U} = 2.4$ µm/s. To assess the assumption that inertial forces are negligible compared to viscous forces, we also compute the Reynolds number

$$\begin{aligned} \text{Re} = \frac{\mathrm {inertial \ forces}}{\mathrm {viscous \ forces}} = \frac{\rho U_r L_r}{\mu }, \end{aligned}$$

with $L_r = 110$ µm (the height of the middle ventricle) and $U_r = 27.9$ µm/s (the maximum velocity magnitude) as length and velocity scales characteristic of the flow.

When studying how cilia paralysis and ventricular morphology affect brain ventricular flow and transport, we calculate the magnitude of a tangential force *F* exerted by the cilia on a part $\mathcal {S}\subset \Gamma _{\text{c}}$ of the ventricular surface as:

6 $$\begin{aligned} F = \int _{\mathcal {S}}\sqrt{\varvec{\tau }\cdot \varvec{\tau }}\,\text{ds}. \end{aligned}$$

### Numerical approximation of the Stokes equations

The Stokes equations (Equation (1)) are discretized with a finite element method in space, while in time, we employ a first-order implicit Euler discretization [105]. We set the timestep size $\Delta t = 0.023$ s such that 20 timesteps represent one cardiac cycle. For the spatial discretization, we use piecewise linear Brezzi-Douglas-Marini (BDM) elements for the velocity. [87, 106] The BDM elements degrees of freedom are associated with integral moments of the velocity component normal to the facets of elements, thus we enforce the impermeability condition $\varvec{u}\cdot \varvec{n}=0$ strongly. We impose the boundary conditions for both the tangential traction $\hat{\boldsymbol{\sigma }}_{\parallel }$ and the normal pressure $\tilde{p}(t)$ weakly. For the pressure, we use piecewise constant (zeroth order discontinuous Galerkin (DG)) elements. The resulting total number of degrees of freedom at each time step is 1 009 874. The element combination $\text{BDM}_1$–$\text{DG}_{0}$ yields a non-conforming discretization scheme for the Stokes equations [107]. In particular, with BDM only the normal component of the velocity is continuous. Continuity in tangential velocities on interior facets is weakly enforced with a penalty parameter $\gamma =10$ [108]. Since the divergence of the velocity function space is the pressure function space for this scheme, the divergence-free condition of the velocity is satisfied exactly, ensuring mass conservation [109].

### Numerical approximation of the advection–diffusion equation

The advection–diffusion equation (Equation (2)) is discretized in space with quadratic DG elements using a symmetric interior penalty DG method [110]. The scheme was chosen due to its favorable mass conservation properties. At each timestep the total number of degrees of freedom is 1 415 120. In time, the equation is discretized with the second-order backward difference scheme BDF2 [111]. To accurately approximate the solute concentrations even in the presence of strong advection (high Péclet numbers), we use an upwind scheme for the velocity when calculating the advective flux [112]. As for the flow equations, we use a timestep size of $\Delta t = 0.023$ s. We simulate transport for 1900 cardiac cycles, with a final time of simulation $T = 856$ s. A DG penalty parameter $\alpha = 50$ was chosen to ensure stability of the method.

### Solution strategy and software implementation

Owing to the one-way coupling between the velocity field $\varvec{u}$ and the concentration *c*, the governing equations for the CSF flow and the concentration can be solved sequentially. For the cilia-driven/no-cardiac flow scenario, the velocity quickly reaches steady-state since the viscous forces dominate the inertial forces. In this case, we first solve the steady-state Stokes equations (Equation (1)) for $\varvec{u}$ and *p*, and then use the velocity field $\varvec{u}$ as input when solving the advection–diffusion equation (Equation (2)). For the other flow scenarios, the Stokes equations are solved for one period of the pulsatile cardiac motion. The solution of this one period is then used periodically as input for the advection–diffusion equation.

The numerical methods for the Stokes equations and the advection–diffusion equation were implemented and solved with DOLFINx [97]. The simulation code is openly available [93]. A direct solver using MUMPS [113] is used to solve the linear systems resulting from the discretized Stokes equations. For the discretized advection–diffusion equation, we use a flexible GMRES solver with block Jacobi preconditioning [114, 115]. We discretize the time-dependent diffusion equation (without advection) and use the assembled linear system matrix as the preconditioner matrix for the advection–diffusion problem. Numerical experiments verified the accuracy of the model implementation (Appendix B).

## Supplementary Information

Supplementary material 1.

Supplementary material 2.

Supplementary material 3.

## Figures and Tables

**Fig. 7:**
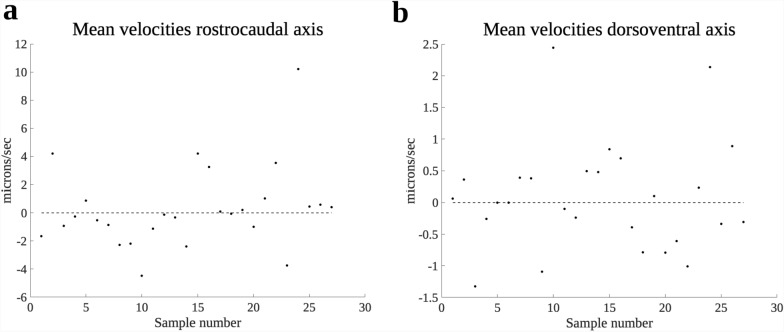
Particle tracking data. Mean velocities in along the rostrocaudal axis (*x* axis, **a**) and along the dorsoventral axis (*z* axis, **b**) directions of the particles tracked. The dashed horizontal lines designate zero

## Data Availability

The software and data used and presented in this paper, as well as the supplementary videos, are archived on Zenodo and openly available at https://doi.org/10.5281/zenodo.15194757.

## Appendix

### A Supplementary theory and numerical methods

#### A.1 Weak formulation of the Stokes equations

We consider the time-dependent Stokes equations

7a $$\begin{aligned} \rho \frac{\partial \varvec{u}}{\partial t} - \nabla \cdot \boldsymbol{\sigma }(\varvec{u}, p)&= \textbf{0} \quad & \text{in} \ \Omega \times (0, T], \end{aligned}$$

7b $$\begin{aligned} \nabla \cdot \varvec{u}&= 0 \quad & \text{in} \ \Omega \times (0, T], \end{aligned}$$

in the domain $\Omega$ with boundary $\Gamma$, where $\boldsymbol{\sigma }(\varvec{u}, p) = 2\mu \boldsymbol{\varepsilon }(\varvec{u}) - p\textbf{I}$ is the viscous stress tensor, in which $\boldsymbol{\varepsilon }(\varvec{u}) = \frac{1}{2}(\nabla \varvec{u}+ (\nabla \varvec{u})^T)$ is the symmetric gradient. Furthermore, $\varvec{u}\in \varvec{V}$ is the fluid velocity and $p\in Q$ is the fluid pressure, and $\rho$ and $\mu$ are the fluid density and dynamic viscosity, respectively. Unless otherwise stated, we let $\boldsymbol{\sigma }= \boldsymbol{\sigma }(\varvec{u}, p)$.

To derive weak formulations of the continuous problem defined by the Stokes equations (Equation (7)), we consider the function space^1^

$$\begin{aligned} \varvec{V}&= \bigg \{\varvec{v}\ : \ \varvec{v}\in \textbf{H}^1(\Omega ), \varvec{v}\cdot \varvec{n}=0 \ \text{on} \ \Gamma \setminus \Gamma _{\text{p}}\bigg \}, \end{aligned}$$

for the velocity and $Q=L^2(\Omega )$ for the pressure. Here $\Gamma _{\text{p}}$ is the anterior/posterior boundary where we impose a normal pressure boundary condition for the model versions that consider fluid motion induced by cardiac pulsations. Bold-faced characters for a space denotes a *d*–dimensional vector space, where *d* is the spatial dimension of $\Omega$. For example, $\textbf{H}^1(\Omega ) = [H^1(\Omega )]^3$ if $\Omega$ is three-dimensional.

To derive a weak formulation of the Stokes equations (Equation (7)), we begin by multiplying Equation (7a) with a test function $\varvec{v}\in \varvec{V}$ and integrating over the domain:

$$\begin{aligned} \int _{\Omega }\rho \frac{\partial \varvec{u}}{\partial t}\cdot \varvec{v} \, \text{d}\varvec{x} - \int _{\Omega }(\nabla \cdot \boldsymbol{\sigma })\cdot \varvec{v} \, \text{d}\varvec{x} = \textbf{0}. \end{aligned}$$

Performing integration by parts of the stress term and applying Green’s first identity, we get

8 $$\begin{aligned} &\int _{\Omega }\rho \frac{\partial \varvec{u}}{\partial t}\cdot \varvec{v} \, \text{d}\varvec{x} - \int _{\Gamma }\boldsymbol{\sigma }\varvec{n}\cdot \varvec{v} \, \text{ds} \\ + &\int _{\Omega }2\mu \boldsymbol{\varepsilon }(\varvec{u}) : \boldsymbol{\varepsilon }(\varvec{v}) \, \text{d}\varvec{x} - \int _{\Omega }p\nabla \cdot \varvec{v} \, \text{d}\varvec{x} = \textbf{0}, \end{aligned}$$

where the symbol: signifies the matrix inner product, $A:B=\text {trace}{(A^TB)}$ for matrices $A, B\in \mathbb {R}^{d\times d}$.

Further steps depend on the boundary conditions applied, which is different for the three model versions we consider in this paper. Before proceeding, let us introduce some notation used in defining the slip boundary conditions. We make use of a decomposition of any vector $\varvec{v}$ on the boundary $\Gamma$ into its normal and tangential components, $\varvec{v}= \varvec{v}_{\perp } + \varvec{v}_{\parallel }$, where $\varvec{v}_{\perp }=(\varvec{v}\cdot \varvec{n})\varvec{n}$ and $\varvec{v}_{\parallel }=P_{\varvec{n}}(\varvec{v})$. The tangential projection is defined as

9 $$\begin{aligned} P_{\varvec{n}}(\varvec{v}) = (\textbf{I} - \varvec{n}\otimes \varvec{n})\varvec{v}, \end{aligned}$$

where $\varvec{n}$ is the outer unit normal of the surface. Introducing $\hat{\boldsymbol{\sigma }} = \boldsymbol{\sigma }\varvec{n}$ for the traction (vector), the forcing due to cilia is represented by constraining $\hat{\boldsymbol{\sigma }}_{\parallel }$ on the part of the boundary populated by cilia. In particular, we let $\hat{\boldsymbol{\sigma }}_{\parallel }= \varvec{\tau }(\varvec{x}, \varvec{r}) = \tau \lambda (\varvec{x}) P_{\varvec{n}}(\varvec{r})$, where the parameter $\tau =0.65$ mPa was used to control the magnitude of the tangential stresses, and chosen by calibrating simulated velocity fields with experimental data [14]. The details of the function $\lambda (\varvec{x})$ follow subsequent to deriving the weak form.

We choose $\varvec{r}= \varvec{r}^+ = (1, 0, 1)$ or $\varvec{r}= \varvec{r}^- = -(1, 0, 1)$ to impose forces perpendicular to the *y*-axis. The reason for this is that all of the experimental images we compare with are taken in the *xz*-plane (rostrocaudal-dorsoventral plane), rendering flow features in the *y*-direction (lateral) unobservable. In the following, we consider the way of handling the stress boundary integral for each model version successively.

**Cilia-driven/no-cardiac model.** This model version has cilia forces as the only mechanism driving flow. We consider the Stokes equations Equation (7) with the slip boundary conditions

10a $$\begin{aligned} \varvec{u}\cdot \varvec{n}&= 0 \quad & \text{on} \ \Gamma =\Gamma _{\text{c}}\cup \Gamma _{\text{s}}, \end{aligned}$$

10b $$\begin{aligned} \hat{\boldsymbol{\sigma }}_{\parallel }&= \varvec{\tau }\quad & \text{on} \ \Gamma _{\text{c}}, \end{aligned}$$

10c $$\begin{aligned} \hat{\boldsymbol{\sigma }}_{\parallel }&= \textbf{0} \quad & \text{on} \ \Gamma _{\text{s}}, \end{aligned}$$

on the boundary $\Gamma =\Gamma _{\text{c}}\cup \Gamma _{\text{s}}$. The boundary $\Gamma _{\text{s}}$ is a free-slip boundary, where we impose the free-slip condition given by Equation (10c). The first condition (Equation (10a)) is the impermeability condition, enforcing no flow normal to the boundary, whereas Equation (10b) imposes a traction tangential to the boundary. This tangential traction is applied to represent the forces that the collective cilia motion exerts on the cerebrospinal fluid.

The boundary conditions defined by Equation (10) are set weakly through the boundary integral

11 $$\begin{aligned} \int _{\Gamma }\boldsymbol{\sigma }\varvec{n}\cdot \varvec{v} \, \text{ds} = \int _{\Gamma }\hat{\boldsymbol{\sigma }}\cdot \varvec{v} \, \text{ds} \end{aligned}$$

in the weak form of the Stokes equations (Equation (8)). We decompose the traction vector $\hat{\boldsymbol{\sigma }}$ into a normal and tangential component:

$$\begin{aligned} \hat{\boldsymbol{\sigma }} = \hat{\boldsymbol{\sigma }}_{\perp }+ \hat{\boldsymbol{\sigma }}_{\parallel }. \end{aligned}$$

Inserting the above into Equation (11), combined with application of the tangential traction condition (Equation (10b)) and the free-slip condition (Equation (10c)), yields

12 $$\begin{aligned} \int _{\Gamma }\hat{\boldsymbol{\sigma }}\cdot \varvec{v} \, \text{ds}= & \int _{\Gamma _{\text{c}}\cup \Gamma _{\text{s}}}{(\hat{\boldsymbol{\sigma }}_{\perp }+ \hat{\boldsymbol{\sigma }}_{\parallel })\cdot \varvec{v}}\, ds = \int _{\Gamma _{\text{c}}\cup \Gamma _{\text{s}}}{\hat{\boldsymbol{\sigma }}_{\perp }\cdot \varvec{v}}\, ds + \int _{\Gamma _{\text{c}}}\varvec{\tau }\cdot \varvec{v} \, \text{ds} \nonumber \\= & \int _{\Gamma }\hat{\boldsymbol{\sigma }}_{\perp }\cdot \varvec{v} \, \text{ds} + \int _{\Gamma _{\text{c}}}\varvec{\tau }\cdot \varvec{v} \, \text{ds}. \end{aligned}$$

Since $\varvec{\tau }$ is known, the second term on the right-hand side of Equation (12) is moved to the right-hand side of the weak form. With $\hat{\boldsymbol{\sigma }}_{\perp } = (\hat{\boldsymbol{\sigma }}\cdot \varvec{n})\varvec{n}$, we can write the first term on the right-hand side of Equation (12) as

13 $$\begin{aligned} \int _{\Gamma }\hat{\boldsymbol{\sigma }}_{\perp }\cdot \varvec{v} \, \text{ds} = \int _{\Gamma }((\hat{\boldsymbol{\sigma }}\cdot \varvec{n})\varvec{n})\cdot \varvec{v} \, \text{ds} = \int _{\Gamma }(\hat{\boldsymbol{\sigma }}\cdot \varvec{n})(\varvec{v}\cdot \varvec{n}) \, \text{ds}. \end{aligned}$$

This term vanishes owing to the construction $\varvec{V}$. As a result, for the cilia-driven/no-cardiac model, the weak form of the momentum equation takes the following form:

$$\begin{aligned} & \int _{\Omega }\rho \frac{\partial \varvec{u}}{\partial t}\cdot \varvec{v} \, \text{d}\varvec{x} + \int _{\Omega }2\mu \boldsymbol{\varepsilon }(\varvec{u}): \boldsymbol{\varepsilon }(\varvec{v}) \, \text{d}\varvec{x} - \int _{\Omega }p\nabla \cdot \varvec{v} \, \text{d}\varvec{x} = \int _{\Gamma _{\text{c}}}\varvec{\tau }\cdot \varvec{v} \, \text{ds}, \\ & \quad \forall \varvec{v}\in \varvec{V}. \end{aligned}$$

Next, multiplying the continuity equation (Equation (7b)) with a test function $q\in Q$, we require that

14 $$\begin{aligned} -\int _{\Omega }q\nabla \cdot \varvec{u} \, \text{d}\varvec{x} = 0,\quad \forall q\in Q. \end{aligned}$$

The full weak form of the Stokes equations for this flow model is then: find $(\varvec{u}, \, p) \in \varvec{V}\times Q$, such that

15a $$\begin{aligned}&\int _{\Omega }\rho \frac{\partial \varvec{u}}{\partial t}\cdot \varvec{v} \, \text{d}\varvec{x} + \int _{\Omega }2\mu \boldsymbol{\varepsilon }(\varvec{u}) : \boldsymbol{\varepsilon }(\varvec{v}) \, \text{d}\varvec{x}- \int _{\Omega }p\nabla \cdot \varvec{v} \, \text{d}\varvec{x}\nonumber \\&\quad = \int _{\Gamma _{\text{c}}}\varvec{\tau }\cdot \varvec{v} \, \text{ds}, \quad \forall \varvec{v}\in \varvec{V}, \end{aligned}$$

15b $$\begin{aligned}&-\int _{\Omega }q\nabla \cdot \varvec{u} \, \text{d}\varvec{x} = 0, \quad \forall q\in Q. \end{aligned}$$

We observe that, in this case with slip boundary conditions on the entirety of the boundary $\Gamma$, for any real constant *C* the vector $(\varvec{u}, p)=(\textbf{0}, C)$ satisfies the equations. Hence Equation (15) is a singular system with a one-dimensional nullspace of constant pressures. Consequently, assembly of the discretization of the Stokes equations (Equation (15)) into a linear equation system $\textbf{A}\textbf{x}=\textbf{b}$ yields a singular matrix $\textbf{A}$. To ensure existence of solutions, we orthogonalize the right-hand side vector $\textbf{b}$ with respect to the pressure nullspace basis (the set of all constants), before solving the linear system. To obtain a unique solution, which together with existence renders the problem well-posed, information of the pressure nullspace is passed to the linear solver. Let us remark that the singularity is a consequence of the fact that the impermeability condition is assumed to hold on the *entire* domain boundary for this model version, which leaves the pressure under-determined as it is not subject to any boundary condition.

For the tangential traction $\varvec{\tau }= \tau \lambda (\varvec{x}) P_{\varvec{n}}(\varvec{r})$ that represents the cilia motion, we heuristically determined the constant $\tau = 0.65$ mPa and the spatially varying function $\lambda (\varvec{x})$. The definitions of $\varvec{r}$ and $\lambda (\varvec{x})$ for the different cilia populations are summarized in Table 2.

**Table 2:** Definitions of $\varvec{r}$ and $\lambda (\varvec{x})$ used in the cilia tangential traction boundary condition $\varvec{\tau }= \tau \lambda (\varvec{x})P_{\varvec{n}}(\varvec{r})$, where $\tau =0.65$ mPa

Cilia population	$\varvec{r}$	$\lambda (\varvec{x})$
Anterior region, anterior ventricle	$-(1, 0, 1)$	$\frac{2}{5}$
Posterior region, anterior ventricle	$\ \ \ (1, 0, 1)$	1
Dorsal region, middle ventricle	$-(1, 0, 1)$	$\frac{11}{4}\left( \frac{x-x_{0, d}}{x_{e, d} - x_{0, d}}\right)$
Ventral region, middle ventricle	$\ \ \ (1, 0, 1)$	$\frac{1}{2}\left( 1 - \frac{x-x_{0, v}}{x_{e, v} - x_{0, v}}\right)$

The $x-$coordinates $x_{0, i}$ and $x_{e, i}$ define the cilia domain in the dorsal ($i=d$) and ventral ($i=v$) regions of the middle ventricle

**Cardiac-induced/no-cilia model.** For this model version, the only physical mechanism inducing fluid flow is the cardiac cycle. The cardiac cycle generates directional back-and-forth motion of the CSF in the zebrafish brain ventricles. We model the influence of the cardiac cycle with a normal pressure boundary condition:

16a $$\begin{aligned} \varvec{u}\cdot \varvec{n}= 0, \ \hat{\boldsymbol{\sigma }}_{\parallel }&= \textbf{0} \quad & \text{on} \ \Gamma _{\text{s}}, \end{aligned}$$

16b $$\begin{aligned} (\mu \nabla \varvec{u}- p\textbf{I})\varvec{n}&= \tilde{p}(t)\varvec{n}\quad & \text{on} \ \Gamma _{\text{p}}, \end{aligned}$$

where the boundary $\Gamma = \Gamma _{\text{s}}\cup \Gamma _{\text{p}}$ consists of a free-slip boundary $\Gamma _{\text{s}}$, and a part $\Gamma _{\text{p}}$ where a normal traction type of boundary condition is imposed. Note, however, that the transpose velocity gradient term must be included inside the parentheses on the left-hand side of Equation (16b) for the expression to be the stress tensor $\boldsymbol{\sigma }$, thus the left-hand side of Equation (16b) does not constitute the normal traction $\hat{\boldsymbol{\sigma }}_{\perp }$. Because of this, we rather refer to this boundary condition as a normal pressure boundary condition. The boundary $\Gamma _{\text{s}}$ is handled as discussed in the previous section, such that the stress boundary integral in Equation (11) vanishes over $\Gamma _{\text{s}}$. Therefore, we now have

17 $$\begin{aligned}&\int _{\Gamma }\hat{\boldsymbol{\sigma }}\cdot \varvec{v} \, \text{ds} = \int _{\Gamma _{\text{p}}}\hat{\boldsymbol{\sigma }}\cdot \varvec{v} \, \text{ds} = \int _{\Gamma _{\text{p}}}(\mu (\nabla \varvec{u}+ (\nabla \varvec{u})^T) - p\textbf{I})\varvec{n}\cdot \varvec{v} \, \text{ds}\nonumber \\&\quad = \int _{\Gamma _{\text{p}}}\mu (\nabla \varvec{u})^T\varvec{n}\cdot \varvec{v} \, \text{ds} + \int _{\Gamma _{\text{p}}}(\mu \nabla \varvec{u}- p\textbf{I})\varvec{n}\cdot \varvec{v} \, \text{ds}\nonumber \\&\quad = \int _{\Gamma _{\text{p}}}\mu (\nabla \varvec{u})^T\varvec{n}\cdot \varvec{v} \, \text{ds} + \int _{\Gamma _{\text{p}}}\tilde{p}(t)(\varvec{v}\cdot \varvec{n}) \, \text{ds}. \end{aligned}$$

We leave the first term on the left-hand side of Equation (17) in the weak form of the momentum equation. The second term is determined by the boundary condition defined in Equation (16b), and is therefore moved to the right-hand side of the weak form. The weak form of the momentum equation is then

18 $$\begin{aligned}&\int _{\Omega }\rho \frac{\partial \varvec{u}}{\partial t}\cdot \varvec{v} \, \text{d}\varvec{x} + \int _{\Omega }2\mu \boldsymbol{\varepsilon }(\varvec{u}) : \boldsymbol{\varepsilon }(\varvec{v}) \, \text{d}\varvec{x} - \int _{\Omega }p\nabla \cdot \varvec{v} \, \text{d}\varvec{x} \\&\qquad -\int _{\Gamma _{\text{p}}}\mu (\nabla \varvec{u})^T\varvec{n}\cdot \varvec{v} \, \text{ds} \\&\quad =\int _{\Gamma _{\text{p}}}\tilde{p}(t)(\varvec{v}\cdot \varvec{n}) \, \text{ds}, \quad \forall \varvec{v}\in \varvec{V}. \end{aligned}$$

Also considering the weak form of the continuity equation, the weak formulation for the cardiac-induced/no-cilia model version then reads: find $(\varvec{u},\, p)\in \varvec{V}\times Q$ such that Equation (18) and Equation (14) hold for all $(\varvec{v}, \, q)\in \varvec{V}\times Q$. To simulate the back-and-forth motion induced by the cardiac cycle observed in previous work [14], we apply sinusoidal forcing. The boundary $\Gamma _{\text{p}}$ has two separate regions: one located on the anterior ventricle and one on the posterior ventricle (Fig. 1d, blue markers). For the normal pressure boundary condition, we set $\tilde{p}(t) = 0$ on the anterior part and $\tilde{p}(t) = p_c(t) = -A\sin {\omega t}$ on the posterior part. The value of the amplitude *A* was based on heuristics and $\omega = 2\pi f$, where $f=2.22$ Hz is the cardiac cycle frequency.

**Baseline model.** The baseline flow model considers both cilia and cardiac beating as driving mechanisms of fluid flow. We therefore consider the boundary conditions

19a $$\begin{aligned} \varvec{u}\cdot \varvec{n}= 0, \ \hat{\boldsymbol{\sigma }}_{\parallel }&= \varvec{\tau }\quad & \text{on} \ \Gamma _{\text{c}}, \end{aligned}$$

19b $$\begin{aligned} \varvec{u}\cdot \varvec{n}= 0, \ \hat{\boldsymbol{\sigma }}_{\parallel }&= \textbf{0} \quad & \text{on} \ \Gamma _{\text{s}}, \end{aligned}$$

19c $$\begin{aligned} (\mu \nabla \varvec{u}- p\textbf{I})\varvec{n}&= \tilde{p}(t)\varvec{n}\quad & \text{on} \ \Gamma _{\text{p}}, \end{aligned}$$

where $\Gamma = \Gamma _{\text{c}}\cup \Gamma _{\text{s}}\cup \Gamma _{\text{p}}$ is the boundary of $\Omega$. The quantities $\varvec{\tau }$ and $\tilde{p}(t)$ have been introduced in the previous sections covering the other flow model versions. We handle the boundary conditions Equation (19a) and Equation (19b) as introduced for the cilia-driven/no-cardiac model, and the boundary condition Equation (19c) as introduced for the cardiac-induced/no-cilia model. The abstract weak formulation for the baseline model is then: find $(\varvec{u},\, p)\in \varvec{V}\times Q$ such that

20a $$\begin{aligned} \mathcal {A}(\varvec{u}, \varvec{v}) + \mathcal {B}(\varvec{v}, p)&= \mathcal {L}(\varvec{v}), & \quad \forall \varvec{v}\in \varvec{V}, \end{aligned}$$

20b $$\begin{aligned} \mathcal {B}(\varvec{u}, q)&= 0, & \quad \forall q\in Q, \end{aligned}$$

with the definitions

21 $$\begin{aligned} \mathcal {A}(\varvec{u}, \varvec{v})&= \int _{\Omega }\rho \frac{\partial \varvec{u}}{\partial t}\cdot \varvec{v} \, \text{d}\varvec{x} + \int _{\Omega }2\mu \boldsymbol{\varepsilon }(\varvec{u}) : \boldsymbol{\varepsilon }(\varvec{v}) \, \text{d}\varvec{x} \nonumber \\&-\int _{\Gamma _{\text{p}}}\mu (\nabla \varvec{u})^T\varvec{n}\cdot \varvec{v} \, \text{ds}, \end{aligned}$$

22 $$\begin{aligned} \mathcal {B}(\varvec{u}, q)&= -\int _{\Omega }q\nabla \cdot \varvec{u} \, \text{d}\varvec{x}, \end{aligned}$$

23 $$\begin{aligned} \mathcal {L}(\varvec{v})&= \int _{\Gamma _{\text{c}}}\varvec{\tau }\cdot \varvec{v} \, \text{ds} + \int _{\Gamma _{\text{p}}}\tilde{p}(t)(\varvec{v}\cdot \varvec{n}) \, \text{ds}. \end{aligned}$$

#### A.2 Discretization of the Stokes equations

We outline the discretization of the weak formulation of the Stokes equations for the baseline model (both cilia traction and cardiac pressure pulsations present) given by Equations (20) to (23). A similar approach can be used to discretize the weak formulation for the other model versions. First, we consider the spatial discretization of the weak formulation. We rewrite the weak form in terms of the discretized variables, denoted with the subscript *h*, on the discrete domain $\Omega _h$:

24a $$\begin{aligned} \mathcal {A}(\varvec{u}_h, \varvec{v}_h) + \mathcal {B}(\varvec{v}_h, p_h)&= \mathcal {L}(\varvec{v}_h), & \quad \forall \varvec{v}_h\in \varvec{V}_h, \end{aligned}$$

24b $$\begin{aligned} \mathcal {B}(\varvec{u}_h, q_h)&= 0, & \quad \forall q_h\in Q_h. \end{aligned}$$

Our choice of discretization are finite elements of the Brezzi-Douglas-Marini (BDM) family [87, 106] for the velocity space and discontinuous Lagrange polynomial elements for the pressure. We refer to the latter as discontinuous Galerkin (DG) elements. For all cells *K* of the computational mesh $\Omega _h$, we define

$$\begin{aligned} \varvec{V}_h&= \Big \{\varvec{v}_h : \varvec{v}_h\in \textbf{H}(\text{div}); \varvec{v}_h\vert _K\in \big [P_k(K)\big ]^d; \forall K\in \Omega _h; \varvec{v}_h\cdot \varvec{n}\\&\qquad =0 \ \text {on } \Gamma _h\setminus \Gamma _{h, \text{p}}\Big \},\\ Q_h&= \Big \{q_h : q_h\in L^2; q_h\vert _K\in P_{k-1}(K); \forall K\in \Omega _h\Big \}, \end{aligned}$$

where $P_k$ is the space of polynomials of degree less than or equal to *k*. The BDM family of elements have degrees of freedom associated with integral moments of vector components normal to facets of the mesh. Because $\varvec{V}_h\not \subset \varvec{V}$, this BDM-DG scheme yields a non-conforming method for the Stokes equations. The scheme is $\textbf{H}(\text{div})$-conforming and exactly mass-conserving: due to the fact that $\nabla \cdot \varvec{V}_h= Q_h$, the continuity equation (Equation (7b)) is satisfied point-wise. Moreover, control of normal components of $\varvec{u}_h$ conveniently lets us enforce the impermeability condition $\varvec{u}_h\cdot \varvec{n}=0$ strongly.

The elements of $\varvec{V}_h$ only ensure by construction the continuity of normal components of $\varvec{u}_h$. Continuity of the tangential components of $\varvec{u}_h$ on interior facets of the mesh is enforced weakly by additional stabilization to the discrete weak form (Equation (24)). Specifically, we apply the stabilization outlined by Hong et al. [108]. First, we define the average and jump operators, respectively, as

25 $$\begin{aligned} \{\varvec{u}\} = \frac{\varvec{u}^+\cdot \varvec{n}^+ - \varvec{u}^-\cdot \varvec{n}^-}{2} \qquad \text{and}\qquad \llbracket \varvec{u}\rrbracket = \varvec{u}^+ - \varvec{u}^-, \end{aligned}$$

where $\varvec{u}$ is a first-order tensor and $\varvec{n}$ is the normal vector on a facet separating two cells, and the plus and minus signs denote variables in each of the two cells. The normal vector is chosen such that it points towards the exterior of its corresponding cell. On the boundary, the operators are defined as $\{\varvec{u}\} = \varvec{u}\cdot \varvec{n}$ and $\llbracket \varvec{u}\rrbracket = \varvec{u}$, with $\varvec{n}$ pointing to the exterior of the domain. Note that average operator defined in Equation (25) reduces the rank of the tensor $\varvec{u}$ by one, whereas the jump operator preserves rank. This extends to tensors of higher order. We now define and add the bilinear form

$$\begin{aligned} \mathcal {A}_{\text{stab}}(\varvec{u}_h, \varvec{v}_h)&= \sum _{f\in \mathcal {F}}\int _f{2\mu \frac{\gamma }{\{h\}}\llbracket P_{\varvec{n}}(\varvec{u}_h)\rrbracket \cdot \llbracket P_{\varvec{n}}(\varvec{v}_h)\rrbracket }\,\text{ds}\\&-\sum _{f\in \mathcal {F}}\int _f{2\mu \{\boldsymbol{\varepsilon }(\varvec{u}_h)\}\cdot \llbracket \varvec{v}_h\rrbracket }\,\text{ds} \\&-\sum _{f\in \mathcal {F}}\int _f{2\mu \{\boldsymbol{\varepsilon }(\varvec{v}_h)\}\cdot \llbracket \varvec{u}_h\rrbracket }\,\text{ds} \end{aligned}$$

to the discrete weak formulation (Equation (24)), where $\mathcal {F}$ is the set of all interior facets of the computational mesh. The first term of $\mathcal {A}_{\text{stab}}$ penalizes jumps in the tangential components of the velocity across interior facets, where the penalty parameter $\gamma >0$ must be chosen large enough to achieve stability. For appropriate convergence, the penalization term includes a mesh-dependent scaling. In this work, we choose it as the average of the cell diameter of neighboring mesh cells, with *h* defined as the maximal distance between two points in a cell [116]. The other two terms of $\mathcal {A}_{\text{stab}}$ are added to ensure consistency and symmetry of the bilinear form, such that the problem is well-defined.

Including the stabilization outlined above, the semi-discrete weak formulation of the Stokes equations reads: find ($\varvec{u}_h, p_h$)$\in$($\varvec{V}_h, Q_h$) such that

26a $$\begin{aligned}&\mathcal {A}(\varvec{u}_h, \varvec{v}_h) + \mathcal {A}_{\text{stab}}(\varvec{u}_h, \varvec{v}_h) + \mathcal {B}(\varvec{v}_h, p_h) = \mathcal {L}(\varvec{v}_h), \quad \forall \varvec{v}_h\in \varvec{V}_h, \end{aligned}$$

26b $$\begin{aligned}&\mathcal {B}(\varvec{u}_h, q_h) = 0, \quad \forall q_h\in Q_h. \end{aligned}$$

In time, we discretize the weak formulation (Equation (26)) with the implicit Euler method, approximating the time derivative as

$$\begin{aligned} \frac{\partial \varvec{u}}{\partial t} \approx \frac{\varvec{u}^{n+1} - \varvec{u}^n}{\Delta t}, \end{aligned}$$

where *n* denotes the timestep index. All of the other terms in Equation (26) are evaluated at timestep $n+1$. The method is implicit and of first order in time [105]. The fully discrete Stokes equations were solved with the penalty parameter $\gamma =10$.

#### A.3 Weak formulation of the advection–diffusion equation

Recall the strong form of the advection–diffusion equation:

27 $$\begin{aligned} \frac{\partial c}{\partial t} + \nabla \cdot \varvec{J}= 0 \quad \text{in} \ \Omega \times (0, T], \end{aligned}$$

where $\Omega$ is the domain with boundary $\Gamma$, and the total solute flux

$$\begin{aligned} \varvec{J}(c) = c\varvec{u}- D\nabla c, \end{aligned}$$

where $c\in W=H^1(\Omega )$ is the solute concentration and $\varvec{u}\in \varvec{V}$ is the cerebrospinal fluid velocity. We consider $\varvec{J}= \varvec{J}(c)$, unless otherwise indicated. The weak form of Equation (27) is derived by multiplying the equation with a test function $\phi \in W$ and integrating over the domain:

$$\begin{aligned} \int _{\Omega }\frac{\partial c}{\partial t}\phi \, \text{d}\varvec{x} + \int _{\Omega }(\nabla \cdot \varvec{J})\phi \, \text{d}\varvec{x} = 0. \end{aligned}$$

Integrating the second term by parts and applying Green’s first identity yields

28 $$\begin{aligned} \int _{\Omega }\frac{\partial c}{\partial t}\phi \, \text{d}\varvec{x} + \int _{\Gamma }(\varvec{J}\cdot \varvec{n})\phi \, \text{ds} - \int _{\Omega }\varvec{J}\cdot \nabla \phi \, \text{d}\varvec{x} = 0. \end{aligned}$$

The boundary integral of the total flux is handled differently for the three different model versions, depending on the boundary conditions considered. For all model versions, we simulate photoconversion for times in $\Omega _{\text{pc}}\subset \Omega$ by strongly enforcing

29 $$\begin{aligned} c(\varvec{x}, t)=\frac{\log {(1+t/a)}}{\log {(1+T/a)}}, \quad \forall \varvec{x}\in \Omega _{\text{pc}}, \end{aligned}$$

for all times *t* until simulation ends at time *T*. The parameter $a=65$ determines the growth speed of the solute concentration and was chosen such that Equation (29) is similar to the experimental photoconversion curves.

**Cilia-driven/no-cardiac model.** We consider $\Omega$ to be a closed domain with impermeable walls, so that the whole boundary $\Gamma$ constitutes a no-flux boundary $\Gamma _{\text{nf}}$. By imposing $\varvec{J}\cdot \varvec{n}=0$ on $\Gamma =\Gamma _{\text{nf}}$, the weak form defined by Equation (28) is reduced to

$$\begin{aligned} \int _{\Omega }\frac{\partial c}{\partial t}\phi \, \text{d}\varvec{x} - \int _{\Omega }\varvec{J}\cdot \nabla \phi \, \text{d}\varvec{x} = 0, \quad \forall \phi \in W. \end{aligned}$$

Defining the left-hand side as the bilinear form

$$\begin{aligned} a(c, \phi ) = \int _{\Omega }\frac{\partial c}{\partial t}\phi \, \text{d}\varvec{x} - \int _{\Omega }\varvec{J}\cdot \nabla \phi \, \text{d}\varvec{x}, \end{aligned}$$

we get the following abstract weak formulation: find $c\in W$ such that

$$\begin{aligned} a(c, \phi ) = 0, \quad \forall \phi \in W. \end{aligned}$$

**Cardiac-induced/no-cilia model.** When using the normal pressure boundary condition (Equation (16b)) to model pulsatile flow, we have in- and outflow of cerebrospinal fluid and thus advection of *c* on the pressure boundary $\Gamma _{\text{p}}$. Let $\Gamma _{\text{p}}=\Gamma _{\text{in}}\cup \Gamma _{\text{out}}$ and $\Gamma = \Gamma _{\text{nf}}\cup \Gamma _{\text{in}}\cup \Gamma _{\text{out}}$, with

$$\begin{aligned} \Gamma _{\text{in}}&= \Big \{\varvec{x}\in \Gamma : \varvec{u}(\varvec{x}, t)\cdot \varvec{n}(\varvec{x}) < 0 \Big \}, \\ \Gamma _{\text{out}}&= \Big \{\varvec{x}\in \Gamma : \varvec{u}(\varvec{x}, t)\cdot \varvec{n}(\varvec{x}) > 0 \Big \}, \end{aligned}$$

as in- and outflow boundaries, respectively, where we have emphasized that both $\varvec{u}$ and $\varvec{n}$ are spatially dependent variables. With $\varvec{J}\cdot \varvec{n}=0$ on the no-flux boundary $\Gamma _{\text{nf}}$, we have

$$\begin{aligned} \int _{\Gamma }(\varvec{J}\cdot \varvec{n})\phi \, \text{ds} = \int _{\Gamma _{\text{in}}}(\varvec{J}\cdot \varvec{n})\phi \, \text{ds} + \int _{\Gamma _{\text{out}}}(\varvec{J}\cdot \varvec{n})\phi \, \text{ds}. \end{aligned}$$

For the outflow boundary integral over $\Gamma _{\text{out}}$, we impose zero diffusive flux $D\nabla c\cdot \varvec{n}=0$, with the advection term $c(\varvec{u}\cdot \varvec{n})$ simply left in the variational form as an unknown. For the continuous problem this is problematic, since we are lacking a boundary condition to close the problem. In the discrete setting, however, this method of leaving the boundary term has been shown to yield consistent results [117–119]. Intuitively, one can think of this outflow boundary condition as setting the advective flux to being the one consistent with the weak form, for which there is a unique *c* satisfying the equations, with $\varvec{u}$ determined by the Stokes equations.

The flux integral over the inflow boundary $\Gamma _{\text{in}}$ cannot be handled in the same way as the method used for $\Gamma _{\text{out}}$, as this would not preserve coercivity of the weak form. Lacking experimental data both for the magnitude and origin of an inflow boundary flux, we deal with the inflow boundary in an explicit way that resembles an approximation of a periodic boundary condition. At a timestep *n*, when solving the linear system for the concentration $c^{n+1}$ at the next timestep $n+1$, we set the inflow flux $h_{\text{in}}^{n+1} = h_{\text{out}}^{n}$, where the latter is the outflow flux

$$\begin{aligned} h_{\text{out}}^{n} = \varvec{J}^n\cdot \varvec{n}= (c^n\varvec{u}^n - D\nabla c^n)\cdot \varvec{n}\end{aligned}$$

at the previous timestep. In the limit that the timestep $\Delta t\rightarrow 0$, we would be imposing

$$\begin{aligned} \int _{\Gamma _{\text{in}}}(\varvec{J}\cdot \varvec{n}) \, \text{ds} = \int _{\Gamma _{\text{out}}}(\varvec{J}\cdot \varvec{n}) \, \text{ds}. \end{aligned}$$

This implies conservation of solute concentration at each timestep, which is the desired behavior as we endeavor to model the ventricles as a closed system. Note that the total concentration would still increase in time, because we impose the concentration profile in the photoconversion region $\Omega _{\text{pc}}$ (region of interest 1).

Using the introduced approaches of handling the in- and outflow boundaries, the resulting abstract weak formulation for the cardiac-induced/no-cilia model is: find $c\in W$ such that

30 $$\begin{aligned} a(c, \phi ) + b(c, \phi ) = L(\phi ), \quad \forall \phi \in W, \end{aligned}$$

with the definitions

31 $$\begin{aligned} a(c, \phi )&= \int _{\Omega }\frac{\partial c}{\partial t}\phi \, \text{d}\varvec{x} - \int _{\Omega }\varvec{J}\cdot \nabla \phi \, \text{d}\varvec{x}, \end{aligned}$$

32 $$\begin{aligned} b(c, \phi )&= \int _{\Gamma _{\text{out}}}c(\varvec{u}\cdot \varvec{n})\phi \, \text{ds}, \end{aligned}$$

33 $$\begin{aligned} L(\phi )&= \int _{\Gamma _{\text{in}}}h_{\text{in}}\phi \, \text{ds}. \end{aligned}$$

**Baseline model.** Since both free-slip and cilia tangential traction boundaries have the same no-flux boundary condition in the advection–diffusion equation, the weak formulation of the advection–diffusion equation for the baseline model is identical to that of the cardiac-induced/no-cilia model, defined by Equations (30), (32) and (33).

#### A.4 Discretization of the advection–diffusion equation

We outline the discretization of the advection–diffusion weak formulation for the baseline model, defined by Equations (30), (32) and (33). The weak formulation is discretized with a symmetric interior-penalty method using discontinuous Galerkin finite elements [110]. Exchanging the variables in Equation (30) with their discrete counterparts yields the discrete weak form: find $c_h\in W_h$ such that

$$\begin{aligned} a(c_h, \phi _h) + b(c_h, \phi _h) = L(\phi _h), \quad \forall \phi _h\in W_h. \end{aligned}$$

The space $W_h$ is constructed with finite elements with discontinuous Lagrange polynomial basis functions:

34 $$\begin{aligned} W_h = \Big \{w_h : w_h\in L^2; w_h\vert _K\in P_k(K); \forall K\in \Omega _h\Big \}. \end{aligned}$$

These elements allow for discontinuities in the discrete solution across element edges. To ensure a stable discretization with these elements, we add the stabilization

35 $$\begin{aligned} \begin{aligned} a_{\text{stab}}(c_h, \phi _h)&= \sum _{f\in \mathcal {F}}\int _f{D\frac{\alpha }{\{h\}}\llbracket c_h\rrbracket \cdot \llbracket \phi _h\rrbracket }\,\text{ds}\\&+\sum _{f\in \mathcal {F}}\int _f{\left( {c_h \hat{u}_h}|^+ - {c_h \hat{u}_h}|^-\right) \cdot \llbracket \phi _h\rrbracket }\,\text{ds} \\&-\sum _{f\in \mathcal {F}}\int _f{D\{\nabla \phi _h\}\cdot \llbracket c_h\rrbracket }\,\text{ds} -\sum _{f\in \mathcal {F}}\int _f{D\{\nabla c_h\}\cdot \llbracket \phi _h\rrbracket }\,\text{ds}, \end{aligned} \end{aligned}$$

to the discrete weak formulation, where

$$\begin{aligned} \hat{u}_h = \frac{1}{2}\left( \varvec{u}_h\cdot \varvec{n}+ \vert \varvec{u}_h\cdot \varvec{n}\vert \right) \end{aligned}$$

is used to upwind the velocity $\varvec{u}_h$ with respect to a facet. The stabilization parameter $\alpha >0$ must be chosen large enough to achieve stability. In Equation (35), the first two terms penalize jumps in the concentration flux across interior facets, and are needed to preserve coercivity of the bilinear form. The last two terms in $a_{\text{stab}}$ are added to preserve symmetry and consistency of the bilinear form, respectively. The average and jump operators used in Equation (35) are defined, respectively, as

$$\begin{aligned} \{c\} = \frac{c^+ + c^-}{2} \qquad \text{and}\qquad \llbracket c\rrbracket = c^+\varvec{n}^+ + c^-\varvec{n}^-. \end{aligned}$$

By including the stabilization $a_{\text{stab}}(c_h, \phi _h)$, the final semi-discrete weak formulation of the advection–diffusion equation is: find $c_h\in W_h$, such that

36 $$\begin{aligned} a(c_h, \phi _h) + a_{\text{stab}}(c_h, \phi _h) + b(c_h, \phi _h) = L(\phi _h), \quad \forall \phi _h\in W_h. \end{aligned}$$

In time, we discretized Equation (36) with the second-order backward difference scheme BDF2 [111]. This scheme approximates the time derivative as

$$\begin{aligned} \frac{\partial c}{\partial t} \approx \frac{3c^{n+1} - 4c^n + c^{n-1}}{2\Delta t}, \end{aligned}$$

where *n* denotes the timestep index. All of the other terms in Equation (36) are evaluated at timestep $n+1$. The method is implicit and of second order in time. We solved the fully discrete weak formulation of the advection–diffusion equation with the penalty parameter value $\alpha =50$.

### B Verification

#### B.1 Method of manufactured solutions

Let $\hat{\varvec{x}} = (\hat{x}, \hat{y}, \hat{z}) = \frac{1}{L}(x, y, z)$ be the spatial coordinates scaled by the length *L*, which is the length of the computational domain $\Omega$ in the *x*-direction. Letting $\varvec{\phi }$ be the vector-valued function

$$\begin{aligned} \varvec{\phi } = \left( \sin {(\pi (\hat{x} - \hat{y}))}, \sin {(\pi (\hat{y} + \hat{z}))}, \sin {(\pi (\hat{x} - \hat{z}))}\right) \end{aligned}$$

and defining the velocity as

37 $$\begin{aligned} \varvec{u}_{\text{mms}} = \nabla \times \varvec{\phi }, \end{aligned}$$

we have by construction that the velocity is divergence-free, owing to the identity $\nabla \cdot (\nabla \times \varvec{F}) = 0$ for an arbitrary vector field $\varvec{F}$. The pressure was defined as

38 $$\begin{aligned} p_{\text{mms}} = \cos {(\pi (\hat{x} + \hat{y} + \hat{z}))}. \end{aligned}$$

Using the Method of Manufactured Solutions [120], we construct a problem with the Stokes equations where Equation (37) and Equation (38) are the solutions. To this end, we consider the general form of the steady Stokes equations

39a $$\begin{aligned} -\nabla \cdot \boldsymbol{\sigma }&= \varvec{f} \qquad \mathrm {in \ }\Omega , \end{aligned}$$

39b $$\begin{aligned} \nabla \cdot \varvec{u}&= g \qquad \mathrm {\, in \ }\Omega , \end{aligned}$$

with $\boldsymbol{\sigma }(\varvec{u}, p) = 2\mu \boldsymbol{\varepsilon }(\varvec{u}) - p\textbf{I}$ and $\boldsymbol{\varepsilon }(\varvec{u}) = \frac{1}{2}\left( \nabla \varvec{u}+ (\nabla \varvec{u})^T\right)$, and the boundary condition

40 $$\begin{aligned} \varvec{u}&= \varvec{u}_{\text{mms}} \qquad & \mathrm {on \ }\Gamma , \end{aligned}$$

where $\Gamma =\partial \Omega$ is the boundary of the domain. Since the velocity field defined by Equation (37) is divergence-free by construction, $g = 0$. Setting the external force $\varvec{f}=-\nabla \cdot \boldsymbol{\sigma }(\varvec{u}_{\text{mms}}, p_{\text{mms}})$ using Equation (37) and Equation (38), the solutions $\varvec{u}$ and *p* to Equation (39) will be the ones defined by Equation (37) and Equation (38), as long as we enforce equality in the mean pressures, i.e. $\int _{\Omega }(p_h - p_{\text{mms}}) \, \text{d}\varvec{x}=0$. Convergence rates are then readily calculated by comparing the numerical approximations of $\varvec{u}$ and *p* with $\varvec{u}_{\text{mms}}$ and $p_{\text{mms}}$ on meshes of varying grid resolution.

#### B.2 Norms and convergence order

Errors of the velocity $\varvec{u}_h$ and pressure $p_h$ approximated with finite elements were measured in $\textbf{H}^1$ and $L^2$ error norms, respectively. With the exact velocity field $\varvec{u}_{\text{ex}}$, the $\textbf{H}^1$ error semi-norm was calculated as

$$\begin{aligned} \vert \varvec{u}_h - \varvec{u}_{\text{ex}}\vert _{\textbf{H}^1}^2 = \int _{\Omega }\nabla (\varvec{u}_h - \varvec{u}_{\text{ex}}) : \nabla (\varvec{u}_h - \varvec{u}_{\text{ex}}) \, \text{d}\varvec{x}. \end{aligned}$$

The $L^2$ error norms for the pressure were calculated as

$$\begin{aligned} {\vert \vert p_h - p_{\text{ex}}\vert \vert }_{L^2}^2 = \int _{\Omega }(p_h - p_{\text{ex}})^2 \, \text{d}\varvec{x}. \end{aligned}$$

The approximated functions were interpolated onto elements in a space of piecewise-continuous Lagrange polynomials of three polynomial degrees higher than the elements used to approximate the functions, meaning that polynomial degrees 4 and 3 were used for the velocity and pressure errors, respectively. Numerical integration was performed with a Gauss quadrature rule, with the quadrature rule degree equal to the polynomial degree of the integrated finite element functions.

Convergence rates reported in Appendix B.3 were approximated in the following way. Define the error $e_k$ for a given mesh resolution $h_k$. Based on the assumption that the error scales as $e_k \approx C{h_k}^\eta$, where *C* is a constant independent of $h_k$, the convergence order $\eta$ was approximated as

41 $$\begin{aligned} \eta = \frac{\log {(e_k/e_{k+1}})}{\log {(h_k/h_{k+1}})}. \end{aligned}$$

We define the approximation of the infinity norm of a finite element function $\phi = \phi _i\psi _i(\varvec{x})$ as

$$\begin{aligned} {\vert \vert \phi \vert \vert }_{L^{\infty }} = \max _i{\vert \phi _i\vert }. \end{aligned}$$

Here $\psi _i$ are the basis functions and $\phi _i$ are the degrees of freedom defined as point evaluations.

#### B.3 Numerical verification of CSF flow model implementation

A cylinder geometry meshed with varying degrees of refinement was used to perform convergence analysis of the numerical method used to solve the Stokes equations. The cylinder had unit length and a diameter of one half length unit. For each mesh refinement, the edge lengths were approximately halved. Since the refinement was not uniform, we used the ratio of the minimal edge lengths $h_{\text{min}, k}/h_{\text{min}, k+1}$ between two successive refinements when calculating the convergence rate using Equation (41). For the analysis, we used tangential traction boundary conditions on all of the cylinder surface. Using the Method of Manufactured Solutions [120] with the velocity and pressure expressions defined in Appendix B.1 for $\varvec{u}_{\text{ex}}$ and $p_{\text{ex}}$, we calculated the error of the velocity approximation $\varvec{u}_h$ in the $\textbf{L}^2$ norm and $\textbf{H}^1$ semi-norm, and the error of the pressure approximation $p_h$ in the $L^2$ norm (Table 3). Based on the error calculations, we calculated the experimental order of convergence, as defined in Appendix B.3. We observe convergence in all of the reported error norms. Furthermore, we report the error in calculation of the maximum velocities, and observe convergence in this error as well. Finally, we calculated the $L^2$ norm of the divergence $\nabla \cdot \varvec{u}_h$ to verify the property of mass conservation.

**Table 3:** Error norms for the convergence study performed with a cylinder mesh with the cilia-driven/no-cardiac flow model using $\mu =1$

# cells	# dofs	${\vert \vert \varvec{u}_h - \varvec{u}_{\text{ex}}\vert \vert }_{\textbf{L}^2}$	$\vert \varvec{u}_h - \varvec{u}_{\text{ex}}\vert _{\textbf{H}^1}$	${\vert \vert p_h - p_{\text{ex}}\vert \vert }_{L^2}$	$\vert {\vert \vert u_h\vert \vert }_{L^{\infty }} - {\vert \vert u_{\text{ex}}\vert \vert }_{L^{\infty }}\vert$	${\vert \vert \nabla \cdot \varvec{u}_h\vert \vert }_{L^2}$
1140	8652	$2.3 \times 10^{-2}$ (–)	$4.3 \times 10^{-1}$ (–)	$5.6 \times 10^{-1}$ (–)	$2.4 \times 10^{-2}$ (–)	$1.4 \times 10^{-7}$
5568	41190	$9.3 \times 10^{-3}$ (1.59)	$2.8 \times 10^{-1}$ (0.75)	$3.9 \times 10^{-1}$ (0.65)	$5.0 \times 10^{-3}$ (2.75)	$2.8 \times 10^{-8}$
35311	255016	$3.5 \times 10^{-3}$ (1.58)	$1.4 \times 10^{-1}$ (1.11)	$2.1 \times 10^{-1}$ (0.95)	$1.4 \times 10^{-3}$ (2.03)	$7.3 \times 10^{-11}$
260717	1855337	$1.3 \times 10^{-3}$ (1.59)	$6.9 \times 10^{-2}$ (1.12)	$1.1 \times 10^{-1}$ (1.00)	$6.7 \times 10^{-4}$ (1.18)	$1.5 \times 10^{-10}$

For all error norms, convergence rates calculated with Equation (41) are given in parentheses

To verify the CSF flow model implementation with the zebrafish brain ventricles mesh, we compared velocity norms for varying degrees of refinement of the brain ventricles mesh (Table 4). We used the original mesh obtained by meshing the surface geometry using fTetWild [96] (the region around the photoconversion region is not refined on the mesh used for the convergence study), and we refined the original mesh using fTetWild. In the numerical experiments, we used the cilia traction vector $\varvec{\tau }= \tau \lambda (\varvec{x}) P_{\varvec{n}}(\varvec{r})$ defined in Table 2 and $\gamma =10$ for the BDM penalty parameter.

For all meshes, we report the maximum discrete velocity magnitude ${\vert \vert u_h\vert \vert }_{L^{\infty }}$, with $u_h=\sqrt{\varvec{u}_h\cdot \varvec{u}_h}$. We also report ${\vert \vert u_h\vert \vert }_{L^{\infty }}$ scaled by the total force $F=\int _{\Gamma _{\text{c}}}\sqrt{\varvec{\tau }\cdot \varvec{\tau }} \, \text{ds}$ applied on the cilia boundary $\Gamma _{\text{c}}$. We chose this measure using *F* as a scale because the regions of the mesh marked as cilia-populated change slightly with every mesh refinement, owing to the unstructured organization of the mesh cells. These changes in the surface area over which the tangential traction is applied result in changes to ${\vert \vert u_h\vert \vert }_{L^{\infty }}$, since the maximum velocity magnitude is a result of the total amount of force *F* applied on the cilia boundary.

To account for the fact that the volume of the domain $|\Omega |=\int _{\Omega } \, \text{d}\varvec{x}$ also changes slightly with mesh refinement, we report the $\textbf{L}^2$ norm of $\varvec{u}_h$ scaled by $|\Omega |$. Finally, we provide verification of mass conservation by reporting the $L^2$ norms of the divergence $\nabla \cdot \varvec{u}_h$. We remark that the units used in the model implementation was millimeters, which is why Table 4 reports values in units of millimeters, instead of micrometers which has been used elsewhere in the paper.

**Table 4:** Velocity norms under mesh refinement of the brain ventricles mesh using the cilia-driven/no-cardiac flow model

# cells	# dofs	${\vert \vert u_h\vert \vert }_{L^{\infty }}$ $\left[ \frac{\textrm{mm}}{\text{s}}\right]$	$\frac{1}{F}{\vert \vert u_h\vert \vert }_{L^{\infty }}$ $\left[ \frac{1}{\mathrm {Pa\cdot mm\cdot s}}\right]$	$\frac{1}{\vert \Omega \vert }{\vert \vert \varvec{u}_h\vert \vert }_{\textbf{L}^2}$ $\left[ \frac{\textrm{mm}}{\text{s}}\right]$	${\vert \vert \nabla \cdot \varvec{u}_h\vert \vert }_{L^2}$ $\left[ \frac{\mathrm {mm^3}}{\mathrm {s^2}}\right]$
132134	943820	0.026	$3.8 \times 10^{3}$	$2.2 \times 10^{-3}$	$1.9 \times 10^{-16}$
180901	1289881	0.027	$3.7 \times 10^{3}$	$2.2 \times 10^{-3}$	$3.0 \times 10^{-16}$
262598	1870703	0.027	$3.7 \times 10^{3}$	$2.3 \times 10^{-3}$	$6.7 \times 10^{-16}$
395339	2811668	0.028	$3.6 \times 10^{3}$	$2.2 \times 10^{-3}$	$2.0 \times 10^{-14}$
627439	4448761	0.027	$3.5 \times 10^{3}$	$2.2 \times 10^{-3}$	$4.9 \times 10^{-14}$

#### B.4 Numerical verification of solute transport model implementation

To verify implementation of the solute transport model, we consider convergence of times to threshold $\hat{t}_i$ in regions of interest (ROIs) *i* and final mean solute concentration $\overline{c}(t=T) = \int _{\Omega }c(\varvec{x}, T) \, \text{d}\varvec{x} / \vert \Omega \vert$ on different refinements of the zebrafish brain ventricles geometries. We used the same meshes as for the flow model verification (see Appendix B.3). Transport of Dendra2 was simulated with CSF velocities from the baseline flow model and the same parameters as used in the simulations presented in the main body of the text: $\alpha =50$, $T=856$, $\Delta t=0.023$. For ROI 1–4 the time $\hat{t}_i$ is when the mean solute concentration $\overline{c}_i$ first exceeded a threshold of 0.25. For ROI 5 and 6 the threshold was 0.10.

**Table 5:** Times to threshold $\hat{t}_i$ in the six regions of interest *i* and final mean solute concentration $\overline{c}(t=T)$ under mesh refinement of the brain ventricles mesh, for simulations of Dendra2 solute transport using the baseline flow model

# cells	# dofs	$\hat{t}_1$ [s]	$\hat{t}_2$ [s]	$\hat{t}_3$ [s]	$\hat{t}_4$ [s]	$\hat{t}_5$ [s]	$\hat{t}_6$ [s]	$\overline{c}(t=T)$ [–]
132134	1321340	61	199	217	167	213	342	0.524
180901	1809010	61	193	210	161	208	338	0.532
262598	2625980	61	190	208	160	206	336	0.529
395339	3953390	61	187	205	156	204	334	0.543
627439	6274390	61	184	201	152	205	326	0.556

#### B.5 Discussion of numerical approximation and convergence rates

A priori error estimates for the Brezzi-Douglas-Marini and discontinuous Galerkin discretization scheme $\text{BDM}_k$-$\text{DG}_{k-1}$ are naturally derived in a mesh-dependent broken $\textbf{H}^1$-norm for $\varvec{u}$ and the $L^2$-norm for the pressure. The respective error norms are expected to decay with order *k* [121]. For the sake of simplicity, Table 3 reports the velocity errors in the standard $\textbf{H}^1$ (semi)norm. Using polynomial degree $k=1$, we observe linear convergence in the velocity in the $\textbf{H}^1$ error norm and the pressure in the $L^2$ error norm on the cylinder mesh. The BDM-DG scheme is thus of first-order accuracy, which may introduce excessive numerical diffusion. However, for the ventricles meshes, we observe consistent values of the reported quantities (Table 4), verifying the model implementation.

The scheme used for discretization of advection–diffusion equation is second-order accurate in space and time. However, accuracy of the discrete model may be influenced by the approximate periodic boundary condition that we employ on the anterior/posterior boundary. To verify the implementation, we report times to threshold in the six regions of interest, as well as the final mean solute concentration (Table 5) for varying degrees of refinement of the ventricles mesh. We observe consistent values across the meshes. Similar to the flow model being influenced by the slight changes in the size of cilia-populated marked regions with mesh refinement, owing to the unstructured organization of the mesh cells, the photoconversion region (region of interest 1) increases slightly with mesh refinement. This is presumably the cause for the increase in total final concentration and thus shorter times to threshold.

The $\text{BDM}_1$-$\text{DG}_0$ scheme that we employ for the Stokes equations is computationally more expensive than more common approaches based on e.g. Taylor-Hood discretization [82] ($P_2-P_1$ elements) or lower-order enriched elements (e.g. MINI) [83]. We opted for the BDM-DG scheme because of its exactly-mass-conserving property, since numerical experiments proved that a divergence-free velocity field was fundamental to attain a stable discretization of the advection–diffusion equation in advection-dominated regions. First, we attempted to discretize the Stokes equations with Taylor-Hood elements, but with the resulting numerical approximation of the velocity $\varvec{u}_h$, we were unable to achieve stability of the advection–diffusion discretization. Numerical experiments identified non-zero divergence of $\varvec{u}_h$ as the root cause of these stability issues. On the standard computational mesh, the $L^2$ norm of $\nabla \cdot \varvec{u}_h$ was in the order of $10^{-6}$, while the maximum value of the divergence of the velocity was of order unity. On a refined mesh with roughly six times as many computational cells as the standard mesh, the numerical values of ${\vert \vert \nabla \cdot \varvec{u}_h\vert \vert }_{L^2}$ and ${\vert \vert \nabla \cdot \varvec{u}_h\vert \vert }_{L^{\infty }}$ remained practically unchanged. This is a result of the fact that incompressibility is only achieved in a weak sense when employing Taylor-Hood elements. The advective part of the total flux is

$$\begin{aligned} \nabla \cdot (c\varvec{u}) = c(\nabla \cdot \varvec{u}) + \varvec{u}\cdot \nabla c. \end{aligned}$$

In the case that $\nabla \cdot \varvec{u}\ne 0$, a source (or sink) term for the concentration is introduced. This may introduce errors in the numerical approximation of *c* and lead to instabilities and spurious oscillations, especially in the case of advection-dominated transport [84–86]. The $\text{BDM}_1$-$\text{DG}_0$ scheme avoids these issues, since $\nabla \cdot \varvec{u}_h=0$ is satisfied exactly as a result of the element definitions.

In addition to its favorable mass-conserving property, the BDM-DG scheme lets us impose the impermeability condition strongly without any tailored approach, since this boundary condition appears naturally in the weak form of the Stokes equations (Appendix A.1) and the BDM elements control the normal component of the velocity. If instead (for example) Taylor-Hood elements were to be used, the impermeability boundary condition must be handled with for example a Nitsche method [116] or with a Lagrange multiplier [122, 123].

### C Quantification of velocities in particle tracking

Particle tracking data [14] was analyzed to discern the relative contribution of cardiac pulsations to CSF flow, as compared to the contribution of motile cilia. For four zebrafish, five recordings with a few particles for each fish were carried out. The particles tracked were in the duct between the rhombencephalic ventricle and the diencephalic ventricle, where cilia do not greatly impact CSF flow [14]. Frequency of image acquisition was 20.22 Hz. The collected data constituted *x* (rostrocaudal axis) and *z* (dorsoventral axis) positions of the particles at every time instant of image acquisition. The velocities $u_x$ and $u_z$ were determined by calculating the change in positions $\Delta x$ and $\Delta z$ relative to the change in time $\Delta t$ between two subsequent images, $\Delta t$ being equal to the frequency of acquisition. Mean velocities $\overline{u}_x$ (Fig. 7a) and $\overline{u}_z$ (Fig. 7b), which serve as a quantification of particle drift, were calculated by taking the difference between the last and first positions of a particle.

Particle movement varied over the different measurements. The mean of $\overline{u}_x$ was 0.26 µm/s and the mean of $\overline{u}_z$ was 0.08 µm/s. The maximum mean velocities in absolute values were 10.2 µm/s and 2.4 µm/s for $\overline{u}_x$ and $\overline{u}_z$, respectively. We used the cardiac-induced/no-cilia flow model to tune the pressure boundary condition amplitude *A*. The choice of $A=1.5$ mPa was used since: (i) it yields a maximum velocity magnitude of 4.8 µm/s, which is close to the maximum observed in the particle tracking data when rejecting the outlier at around 10 µm/s; (ii) it yields a time-averaged mean velocity of 0.89 µm/s, which is close to the mean observed in the particle tracking data.
