# Supplementary material for: Advection versus diffusion in brain ventricular transport
Source: Fluids Barriers CNS. 2025 Aug 13;22:82. doi: 10.1186/s12987-025-00692-3 (PMC12344991; doi:10.1186/s12987-025-00692-3)
Supplement: Supplementary file 1 — Supplementary material 1. [file 12987_2025_692_MOESM1_ESM.pdf]

<u>Supplemental information</u>

<u>Video S1:</u> CSF flow simulation with motile cilia and cardiac pulsatility. Initial view is lateral (xz-plane). Velocity vectors are scaled by magnitude, and colored by the magnitude signed with the x component, such that rostrocaudal flow is red and caudorostral flow is blue. The video spans 24 cardiac cycles at 22 fps rate (half of real-time).

<u>Video S2:</u> Transport of secreted and photoconverted Dendra2 in the zebrafish ventricle. Dual color time-lapse confocal images show the location of unconverted (green) and converted (magenta) signals over time. Acquisition rate: 2.67s per frame. Photoconversion is performed from frame 11 to the end of the recording (frame 300).
